# Integrin αvβ3 and CD44 pathways in metastatic prostate cancer cells support osteoclastogenesis via a Runx2/Smad 5/receptor activator of NF-κB ligand signaling axis

**DOI:** 10.1186/1476-4598-11-66

**Published:** 2012-09-11

**Authors:** Aditi Gupta, Wei Cao, Meenakshi A Chellaiah

**Affiliations:** 1 Department of Oncology and Diagnostic Sciences, Dental School, University of Maryland, Baltimore, MD, 21201, USA; 2 Department of Oral and Maxillofacial Surgery, Ninth People’s hospital, Shanghai Jiao Tong University School of Medicine, Shanghai, 200011, China

**Keywords:** PC3 cells, RANKL, RUNX2, Smad 5, CD44, Integrin αvβ3, Osteoclasts, PKC

## Abstract

**Background:**

Bone loss and pathological fractures are common skeletal complications associated with androgen deprivation therapy and bone metastases in prostate cancer patients. We have previously demonstrated that prostate cancer cells secrete receptor activator of NF-kB ligand (RANKL), a protein essential for osteoclast differentiation and activation. However, the mechanism(s) by which RANKL is produced remains to be determined. The objective of this study is to gain insight into the molecular mechanisms controlling RANKL expression in metastatic prostate cancer cells.

**Results:**

We show here that phosphorylation of Smad 5 by integrin αvβ3 and RUNX2 by CD44 signaling, respectively, regulates RANKL expression in human-derived PC3 prostate cancer cells isolated from bone metastasis. We found that RUNX2 intranuclear targeting is mediated by phosphorylation of Smad 5. Indeed, Smad5 knock-down via RNA interference and inhibition of Smad 5 phosphorylation by an αv inhibitor reduced RUNX2 nuclear localization and RANKL expression. Similarly, knockdown of CD44 or RUNX2 attenuated the expression of RANKL. As a result, conditioned media from these cells failed to support osteoclast differentiation in vitro. Immunohistochemistry analysis of tissue microarray sections containing primary prostatic tumor (grade2-4) detected predominant localization of RUNX2 and phosphorylated Smad 5 in the nuclei. Immunoblotting analyses of nuclear lysates from prostate tumor tissue corroborate these observations.

**Conclusions:**

Collectively, we show that CD44 signaling regulates phosphorylation of RUNX2. Localization of RUNX2 in the nucleus requires phosphorylation of Smad-5 by integrin αvβ3 signaling. Our results suggest possible integration of two different pathways in the expression of RANKL. These observations imply a novel mechanistic insight into the role of these proteins in bone loss associated with bone metastases in patients with prostate cancer.

## Introduction

Prostate cancer is the most prevalent non-skin cancer to affect men and it is the second leading cause of cancer-related deaths in Western males [1,2]. The majority of the patients with advanced prostate cancer will eventually develop bone metastases [3]. Prostate cancer cells that metastasize to bone have the capacity to produce osteolytic lesions which are due to activation of osteoclasts [4]. Likewise, bone loss is increasingly recognized as a common occurrence in men diagnosed with prostate cancer receiving androgen deprivation therapy (ADT). The receptor activator of nuclear factor kB ligand (RANKL) is an essential cytokine required for the formation and activation of osteoclasts [5-7]. The involvement of RANKL in the progression of prostate tumor growth within bone and the subsequent bone loss has been recently established in animal models of cancer metastasis [8-13].

Runx2, a transcription factor that plays a key regulatory role in osteoblast differentiation, is also highly expressed in bone metastatic breast and prostate cancer cells [14-16]. RUNX2 increases the oncogenic potential through regulation of genes (e.g. MMP2, MMP9, and MMP13) involved in metastasis and invasion of prostate and breast cancer cells [17-19]. RUNX2 expression in cancer cells facilitates the interaction between tumor cells and the bone microenvironment that lead to osteolytic disease [15,20]. For instance, in vivo blockade of the Runx2-Indian hedgehog pathway in MDA-MB-231 cells by targeting Runx2 with short hairpin RNA prevented osteolytic disease [21]. Furthermore, the presence of putative binding sites for RUNX2 in the promoter region of RANKL [22] and a striking decrease in the number of osteoclasts in RUNX2- (Cbfa1-) deficient mice [22] suggest that RUNX2 is potentially involved in RANKL expression.

Smads, a family of proteins involved in the translocation of signals from receptors to the nucleus have been shown to physically interact with RUNX2 [23]. Interaction between these proteins results in the formation of transcriptionally active complexes which hold the potential to regulate various developmental and biological processes [24,25]. In fact, cooperation between Smads and RUNX2 induces osteoblast specific gene expression in mesenchymal stem cells to promote osteoblast differentiation [24,26,27]. The role of RUNX2 and Smads has been extensively studied in a variety of cell systems. However, the combined roles of these proteins and their signaling mechanisms on RANKL expression in bone metastatic prostate cancer cells have been largely unexplored.

Integrin αvβ3 and CD44 signaling have been shown to increase the metastatic potential of cancer cells [28-30]. Integrin αvβ3 expression in tumor cells accelerates the development of osteolytic lesions [31]. Integrin αvβ3 signaling has been implicated in the expression of RANKL and osteoclastogenesis by breast cancer in the bone microenvironment [32]. CD44 signaling increases the metastatic potential of prostate cancer cells [33,34]. Altered levels of CD44 have been seen in many epithelial neoplasms and expression of CD44 has been shown to carry prognostic implications [35,36]. RUNX2 expression is regulated by CD44 signaling [37]. A neutralizing antibody to CD44s significantly decreased the expression of Runx2 mRNA in hypertrophic chondrocytes [37]. CD44 signaling is a determinant of inflammatory bone loss through expression of RANKL [38,39]. PC3 and LNCaP cell lines have been used by many researchers to document the role of CD44 in the metastatic process [40-43]. We have previously demonstrated that osteopontin regulates the expression and secretion of RANKL in PC3 cells [28]. However, the molecular mechanisms underlying the expression of RANKL are not fully understood. The role of multiple receptor signaling pathways (for e.g. CD44 and integrin αvβ3) converge on the transcriptional factor(s) to regulate RANKL expression needs further elucidation.

Therefore, our aim is to further elucidate the mechanisms by which RANKL expression is regulated by testing the hypothesis that integrin αvβ3 and CD44 signaling plays a key role in mediating the expression of RANKL. Understanding the molecular mechanisms underlying RANKL expression may provide a valuable insight into the process of osteoclast differentiation and the resultant bone resorptive activities within the skeletal microenvironment. In the present study, the cooperative role of RUNX2 and Smad5 in the expression of RANKL was studied in PC3 cells. Here, we provide compelling evidence that a) CD44 signaling regulates the phosphorylation of RUNX2; b) CD44 knockdown reduced RUNX2 phosphorylation, but not Smad 5 phosphorylation; c) knockdown of Smad 5 levels or suppression of phosphorylation of Smad 5 by an inhibitor to integrin αv reduced nuclear localization of RUNX2, and d) inhibition of phosphorylation of either RUNX2 or Smad 5 reduces the expression of RANKL and osteoclast differentiation.

## Results

We have primarily used PC3 cells derived from bony metastasis for various analyses. We have also used prostate cancer cells derived from brain (DU145) and lymph node (LNCaP) metastases for comparative analyses. Normal prostatic epithelial (HPR1) and benign prostatic hyperplasic (BPH) cells were used as controls.

### RUNX2 expression is markedly increased in bone metastatic prostate cancer cells

**Figure 1:**
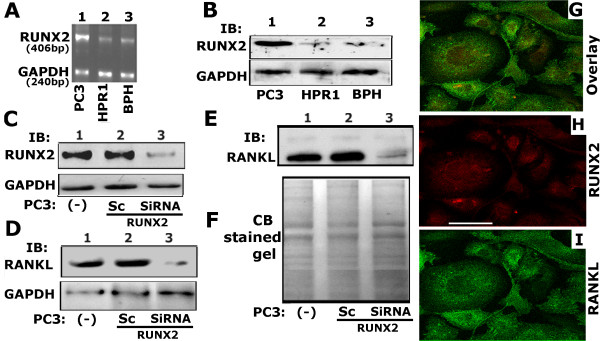
**Analysis of expression of RUNX2 and RANKL in PC3 cells. A** and **B**. RT-PCR and immunoblotting analysis of expression of RUNX2 in PC3 (lanes 1), HPR1 (lane 2) and BPH (lane 3) cells is shown. **C**-**E**: The effects of SiRNA to RUNX2 on RUNX2 (C) and RANKL (D) protein levels in total cellular lysates (C and D) and conditioned medium (E). Immunoblotting analysis in conditioned medium represents the secreted levels of RANKL. Untransfected (−) or scrambled SiRNA (Sc) transfected PC3 cells were used as controls (B-E). GAPDH was used as a loading control for RT-PCR (A) and Western blot (B -D) analyses. The loading control for the conditioned medium is shown by the use of Coomassie blue staining of the blot (F). **G - I**: Immunostaining and confocal microscopy analysis of distribution of RUNX2 (red; H) and RANKL (green; I) in PC3 cells. Distribution of both RANKL (red) and RUNX2 (green) are shown in panel G. Results shown are representative of three independent experiments. Scale bar: 50 μm.

We initially examined the levels of RUNX2 expression in PC3 and control (HPR1 and BPH) cell lines (Figure 1A). RUNX2 expression was considerably higher at mRNA (Figure 1A, lane 1) and protein (B, lane 1) levels as compared with other control cell lines tested (A and B; lanes 2 and 3).

#### RUNX2 ablation reduces RANKL expression

RUNX2 is linked to MMP9 and RANKL expression [44,45]. First, we attempted to determine the efficient dose of SiRNA to RUNX2 to knockdown RANKL. The knockdown of Runx2 by RNA interference decreases MMP9 expression [44]. Therefore, we have assessed the effects of different doses (10, 25, 50nM) of RUNX2 SiRNA nucleotide on the expression of MMP9 and MMP2 at mRNA and protein levels (Additional file 1: Figure S1). RT-PCR analysis demonstrated dose-dependent decrease in the expression of MMP9 at mRNA level and not MMP2. The decrease was maximal at 50nM (>90%; Additional file 1: Figure S1A). A significant decrease in the expression of MMP9 and not MMP2 protein was observed with 50nM SiRNA to RUNX2 (Additional file 1: Figure S1D and E). Therefore, in further experiments, PC3 cells were transfected with 50nM SiRNA nucleotides to RUNX2. Immunoblotting analysis shows the silencing effect >80% at 50nM SiRNA on RUNX2 protein level (Figure 1C, lane 3).

Subsequently, we determined the effects of RUNX2 knockdown on the expression of RANKL in PC3 cells treated with 50nM SiRNA. RUNX2 ablation reduces total cellular and secreted (Figure 1D and E, lane 3) RANKL to a significant level (>85%). Secreted RANKL was determined in the conditioned medium (E and F). Untransfected ((−); C-F, lane 1) and ScSiRNA (50nM; lane 2 in C-F) transfected PC3 cells were used as controls.

### Differential intracellular localization of RANKL and RUNX2 in PC3 cells

We examined the cellular distribution of RANKL and RUNX2 by immunostaining and confocal analyses in PC3 cells (Figure 1G-I). Diffuse and punctate distribution of RANKL (green) and RUNX2 (red) was observed. RUNX2 distribution was observed in the perinuclear and nuclear region. Lateral confocal sectioning and XZ scanning of PC3 cells displayed distribution of RANKL throughout cytoplasm and membrane (data not shown). Colocalization of RANKL and RUNX2 was negligible. Differential subcellular localization of these proteins may be important for their function.

### ChIP analysis of Runx2**-**binding **sites in the RANKL** promoter

**Figure 2:**
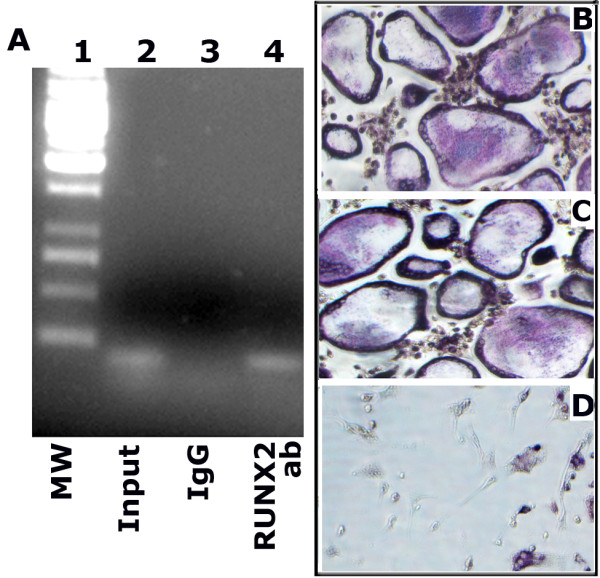
**Analysis of binding of RUNX2 with RANKL promoter and the effect of RUNX2 knockdown on osteoclast differentiation. A**. Chromatin immunoprecipitation (ChIP) assay. ChIP assay was used to determine the RUNX2 binding sites in RANKL promoter. Immunoprecipitates were made with an antibody (rabbit) to RUNX2 (lane 4) or rabbit IgG (lane 3) using lysates made from PC3 cells. DNA from the input (lane 2) and immunoprecipitates (lanes 3 and 4) was analyzed by RT-PCR using primers specific for RUNX2 binding sites on RANKL promoter. As expected, a product size 153 bp was observed in the RT-PCR analysis. The experiment was repeated twice and obtained similar results. **B**-**D**: The conditioned media (CM) from PC3 cells untreated (B) or treated with scrambled (C) and SiRNA (D) to RUNX2 were used for osteoclast differentiation in vitro. TRAP-positive osteoclasts are stained in dark purple. Cells were observed under an inverted phase contrast microscope and images were captured (X 200). The results shown are representative of three experiments.

Two sets of primers specific for RUNX2 binding sites on RANKL promoter were used to detect the DNA fragment (153 bp; Figure 2A, lanes 2 and 4) positioned between nucleotide −143 and −300 in human RANKL promoter [46]. This fragment encompasses the RUNX2 binding site located between −228 to −234 nucleotides. RT-PCR analysis demonstrated the expected product of 153 bp DNA fragment which suggests direct binding of RUNX2 to the RANKL promoter (Figure 2A, lanes 2 and 4). 

### Ablation of RUNX2 reduces osteoclast differentiation

To analyze whether RUNX2 knockdown in PC3 cells would modulate osteoclast differentiation, conditioned media (CM; 50-100 μg protein) from PC3 cells untreated (Figure 2B) or treated with scrambled (C) and SiRNA (D) to RUNX2 were incubated with mouse bone marrow cells in the presence of mCSF1 to induce osteoclast differentiation in vitro. As shown in Figure 2, CM from PC3 cells untransfected (B) or transfected with scrambled SiRNA to RUNX2 induces differentiation of bone marrow cells to mature osteoclasts. Conversely, osteoclast differentiation was prevented by CM from PC3 cells knockdown of RUNX2 (D) suggesting that RUNX2 regulates RANKL expression, and that secretion of RANKL by metastatic prostate cancer cells in the bone microenvironment may support osteoclastogenesis and osteolysis.

### CD44 knockdown reduces RANKL expression and osteoclast differentiation

Our previous observation demonstrated an underlying correlation between osteopontin/CD44 signaling and RANKL expression [28]. CD44 increases RANKL expression in bone marrow stromal cells (BMSCs). BMSCs isolated from CD44 knockout mice express less RANKL [47]. Therefore, we sought to determine in PC3 cells, the possible regulatory mechanisms involved in the activation of RUNX2 and the role of CD44 signaling in this process*.*

#### CD44 is highly expressed in PC3 cells

**Figure 3:**
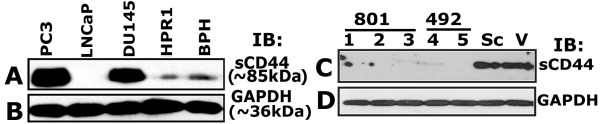
**Characterization of stable CD44 knockdown cell lines. A**. Western blot analysis: Equal amount of protein lysates (50 μg) made from indicated cell lines were immunoblotted with a CD44 antibody to detect total cellular levels of CD44 protein. **C**. Immunoblotting analysis of the total cellular levels of CD44 in the stable clonal isolates derived from PC3 cells transfected with CD44 ShRNA constructs (801 and 492; lanes 1–5) is shown. PC3 cells transfected with vector DNA (V) and scrambled ShRNA construct (Sc) were used as controls. **B** and **D**: Equal loading of protein was verified with the GAPDH level in each lane. The experiment was carried out three times with similar results.

At first, we evaluated the expression levels of CD44 in control cells (HPR1 and BPH) and prostate cancer cells derived from bone (PC3), lymph node (LNCaP) and brain (DU145) metastases (Figure 3A). Expression of CD44 was observed in the following order in the cell lines tested: PC3 >DU145 >BPH = HPR1 (Figure 3A). The blot shown in Figure 3A was exposed for >5 min in order to observe the expression levels of CD44 in LNCaP, BPH and HPR-1 cells. Expression of CD44 was very negligible in BPH and HPR-1 cells. As shown by others, CD44 was not observed in LNCaP cells (Figure 3A) [40,48,49]. 

#### Generation of stable CD44 knockdown PC3 cells

In order to determine the role of CD44 in the expression of RANKL, we have generated PC3 cells knockdown of CD44. Four constructs were made to knockdown CD44 as described in the Methods section. A significant decrease in the expression levels of CD44 was observed in PC3 cells transfected with silencing CD44 ShRNA constructs corresponding to nucleotide sequences 492 bp and 801 bp (Figure 3C). We have generated about 15–20 individual clones and tested for the expression of CD44. The expression levels of standard CD44 in the clonal isolates of 801 (lanes 1–3) and 492 (lanes 4 and 5) ShRNA constructs are shown (Figure 3C). Among the individual clones tested, one clonal isolate which demonstrated maximum knockdown of CD44 from 801 and 492 group (Figure 3C, lanes 1 and 5) was propagated for further studies shown below. Additionally, immunoblot analyses show that these cells are negative for CD44 variant isoforms (data not shown). Non-silencing scrambled ShRNA (Sc) construct and vector DNA (V) transfected cells were used as controls.

#### RANKL expression and osteoclast differentiation is reduced in PC3 cells knockdown of CD44

**Figure 4:**
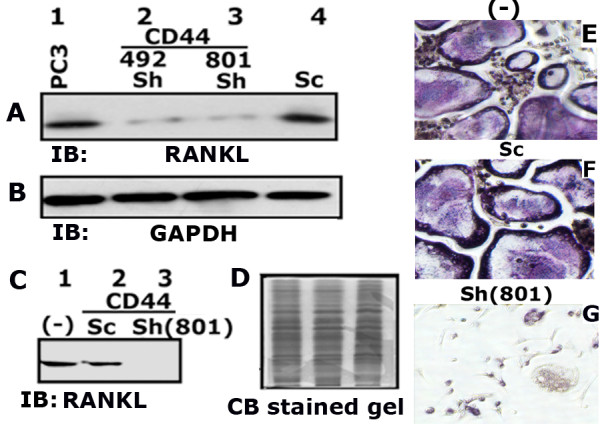
**Analysis of RANKL expression level in PC3 cells knockdown of CD44. A** and **C**. Equal amount of total cellular lysates (50 μg protein; A) and conditioned media (CM-20 μg protein; C) were immunoblotted with a RANKL antibody to detect RANKL protein. CM was used to detect the secreted RANKL protein. **B** and **D**. The blot in A was stripped and reprobed with a GAPDH antibody. Equal level of GAPDH protein was observed (B). The loading control for the CM was shown by the use of Coomassie blue staining of a gel ran in parallel (D). **E**-**G**. The effect of CM on osteoclast differentiation in vitro is shown. TRAP-positive osteoclasts are stained in dark purple. Images were captured (X 200) with an inverted phase contrast microscope. The results shown are representative of three independent experiments.

We subsequently evaluated the total cellular (Figure 4A) and secreted (Figure 4C) levels of RANKL in CD44 knockdown clones and control cells. Secreted levels of RANKL in CM (Figure 4C, lane 3) and the effect of CM on osteoclast differentiation (Figure 4G) were shown with studies carried with a clonal isolate derived from the 801 bp construct. A significant decrease in the cellular (Figure 4A, lanes 2 and 3) and secreted levels (Figure 4C, lanes 3) of RANKL was observed in CD44 knockdown cells (PC3/Sh (801)) as compared with control cells (A, lanes 1 and 4; C, lanes 1 and 2).

CM from PC3/ShCD44 (801) cells failed to support differentiation of mouse bone marrow cells into multinucleated osteoclasts (Figure 4G). Multinucleated giant osteoclasts were observed in bone marrow cultures added with CM media from control PC3 cells (Figure 4E and F). Overall, these results implicate CD44 signaling as an important mediator of RANKL expression.

### CD44 signaling regulates RUNX2 expression

**Figure 5:**
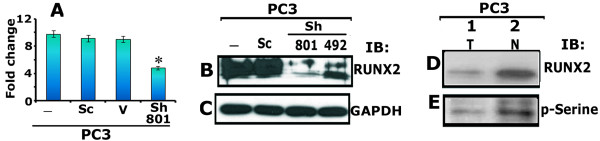
**Effects of CD44 knockdown on RUNX2 expression (mRNA and protein) and distribution in PC3 cells. A**. The expression levels of RUNX2 mRNA was determined by real-time PCR analysis and normalized relative to GAPDH expression. Bar represents the mean ± SEM of three different experiments. *p <0.01 vs. untransfected (−) and transfected PC3 cells with scrambled ShRNA construct (Sc) and vector DNA (V). **B** and **C**. Equal amount of lysates (20 μg protein) made from PC3 cells untransfected (−) and transfected with scramble (Sc) and ShRNA CD44 constructs (492 and 801) were used for immunoblotting analysis with an antibody to RUNX2. Immunoblotting with an antibody to GAPDH (C) was used as a loading control. **D** and **E**. PC3 cells were analyzed for the phosphorylation of RUNX2 in total cellular (T) and nuclear (N) lysates by immunoblotting of RUNX2 immunoprecipitates with antibodies to RUNX2 (D) and phospho-serine (E; p-Serine). The results shown are representative of three independent experiments.

CD44-mediated signaling appears to have a role in the expression of RUNX2 because a neutralizing antibody to CD44 attenuated RUNX2 expression in chondrocytes [39]. Therefore, we examined the functional relationship between CD44 receptor and RUNX2 expression in indicated PC3 cell lines by real-time PCR (Figure 5A) and Western blot (Figure 5B) analyses. Knockdown of CD44 in PC3 cells reduces the expression of RUNX2 at mRNA (Figure 5A; sh801) and protein levels (B, Sh492 and 801) as compared to indicated control cells. 

Previous studies have shown that phosphorylation of RUNX2 occurred mostly on the serine residues with a small amount at threonine and tyrosine residues [19,50]. Therefore, we determined the serine phosphorylation status of RUNX2 (Figure 5E) in PC3 cells. RUNX2 immunoprecipitates from total cellular (T) and nuclear (N) lysates were used for immunoblotting with an antibody to RUNX2 (D) and phospho-Serine (p-Serine; E). Phosphorylation of RUNX2 corresponds with the protein level present in the whole cell and nuclear lysates. Reduced phosphorylation corresponds with the low levels of RUNX2 in whole cell lysates (D and E, lane 1) and the opposite is true for the nuclear lysates (lane 2 in D and E). This result is in agreement with the nuclear localization of RUNX2 in immunostaining analysis (Figure 1G).

### p-Smad 5 localizes in the nuclear region

Several lines of evidence suggest that RUNX2 functions synergistically with a family of Smad proteins to induce osteogenesis and modulate tumor growth and metastasis [51].Therefore, we proceeded to determine whether Smad protein(s) have any synergistic role with RUNX2. First, we analyzed the expression and phosphorylation levels of Smad 2, 3, 5 and 6 in total PC-3 cellular lysates. Our analyses indeed have shown the presence of Smad 2, 3 and Smad 5 proteins and not Smad 6 in PC3 cells. However, we found that the phosphorylation status of Smad 5 was significantly higher than in Smad 2 and 3 (see Additional file 2: Figure S2). Therefore, we decided to focus our attention on the role of Smad 5 in RUNX2 function.

**Figure 6:**
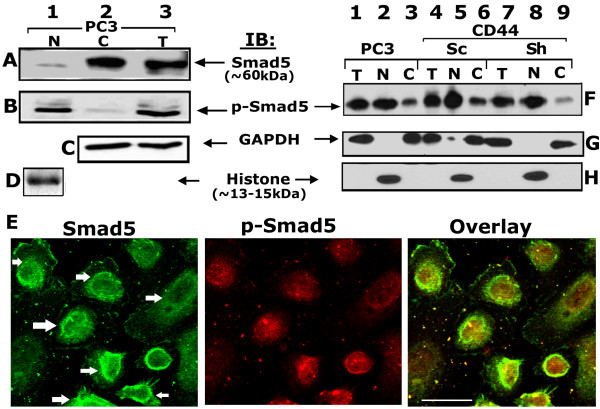
**Analysis of Smad 5 phosphorylation in PC3 cells. A** and **B**; **F**-**H**. Protein and phosphorylation levels of Smad 5 were determined by Western blot analysis in nuclear (N), cytosolic (C) and total cellular (T) proteins isolated from PC3 cells (A and B) and PC3 cells knockdown of CD44 (F-H). 50 μg of indicated protein (A-D, F-H) was used for immunoblotting (IB) analyses. The blot in A was stripped and reprobed successively with p-Smad 5, GAPDH and histone antibodies (B-D). Similarly, the blot in F was stripped and reprobed twice simultaneously with GAPDH and histone antibodies (G and H). Immunoblotting with an antibody to GAPDH (C and G) and histone (D and H) was used as a control for normalization of cellular and nuclear protein, respectively. **E**. Confocal analysis of immunostained PC3 cells with Smad 5 (green) and p-Smad 5 (red) antibodies is shown. Distribution of both Smad 5 and p-Smad 5 is shown in the overlay panel. Scale bar-50 μm. The results shown are representative of three independent experiments.

We first investigated the nuclear (N, 100 μg), cytoplasmic (C, 100 μg) and total cellular (T, 200 μg) levels of Smad 5 (Figure 6 A) and phospho-Smad 5 (p-Smad 5; B) by immunoblotting analyses. Smad 5 was observed predominantly in total cellular (T) and cytosolic (C) lysates (Figure 6A, lanes 2 and 3). However, a significantly lower level of p-Smad 5 was observed in the cytosolic protein (Figure 6B, lane 2). In contrast, equal levels of phosphorylation of Smad 5 was detected in total cellular and nuclear (N) lysates (Figure 6B, lanes 1 and 3) although significantly lower level of Smad 5 was present in the nuclear lysates (A, lane 1). It is possible that the p-Smad 5 recognized in the total cellular lysate (Figure 6B, lane 3) may represent the one present in the nucleus (6B, lane 1).

Immunostaining and confocal microscopy analyses (Figure 6E) corroborated the immunoblotting analysis. Strong Smad 5 staining was observed at the perinuclear region (indicated by arrows in the green panel) with a diffuse distribution in the nuclei. Distribution in the perinuclear region includes the nuclear membrane. Also, Smad 5 was present in the cytoplasm and plasma membrane, but to a lesser extent (Figure 6E; green panel). However, localization of p-Smad 5 was observed largely in the nucleus (Figure 6E, red). Perinuclear distribution of Smad 5 may support the phosphorylation event and immediate export into the nuclei at the time of transcription.

### Phosphorylation of Smad 5 occurs independent of CD44 signaling

To determine the role of CD44 signaling in the phosphorylation of Smad 5, we used the stable PC3/ShCD44 (801) cell line. Phosphorylation of Smad 5 remained the same in total cellular (T) and nuclear (N) protein of PC3 cells untransfected (Figure 6F, lanes 1 and 2) or transfected with scrambled ShRNA (Figure 6F, lanes 4, 5) and ShRNA (lanes 7 and 8) constructs to CD44. Consistently, phosphorylation is significantly lower in the cytosolic protein (lanes 3, 6, and 9) than total cellular (T) and nuclear (N) proteins (Figure 6F). Knockdown of CD44 signaling had no effects on the expression, phosphorylation or nuclear localization of Smad 5 protein (Figure 6F, lanes 7–9). These findings clearly indicate that CD44 signaling appears to have no role in the phosphorylation of Smad 5.

### Phosphorylation of Smad 5 regulates nuclear localization of RUNX2

**Figure 7:**
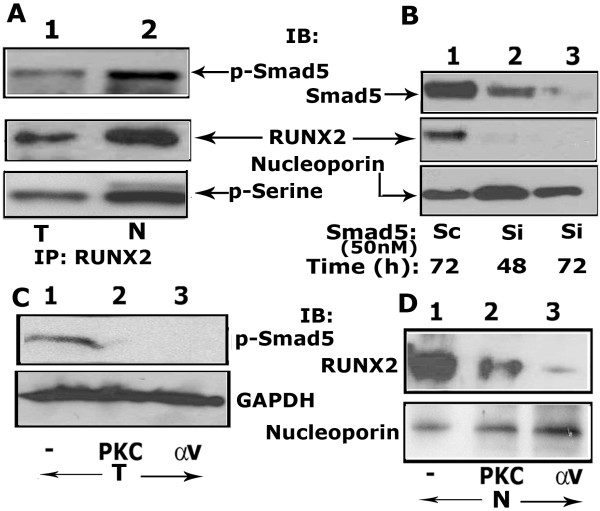
**The effect of PKC and integrin αv inhibitor on the phosphorylation of Smad 5 and RUNX2 localization in the nuclei. A**. Analysis of interaction of p-Smad 5 with RUNX2. Equal amount of total cellular and nuclear proteins were immunoprecipitated with a RUNX2 antibody and immunoblotted with a p-Smad 5 antibody (A, top panel). Subsequently, the blot was reprobed sequentially with a RUNX2 (middle panel) and p-Serine (bottom panel) antibody after stripping. **B**. Effect of SiRNA to Smad 5 on the nuclear levels of RUNX2. Time-dependent effect of SiRNA (Si) nucleotides on Smad 5 levels at 48 and 72 h is shown. Equal amount of nuclear proteins were immunoblotted sequentially with antibodies to Smad 5, RUNX2 and nucleoporin after stripping. Scrambled RNAi nucleotide (Sc) transfected cells were used as controls (lane 1). **C**. Effects of PKC and integrin αv inhibitors (lanes 2 and 3) on the phosphorylation of Smad 5. Untreated (-) PC3 cells were used as control. Total cellular (T) lysate proteins were immunoblotted with a p-Smad 5 antibody. **D**. Effects of PKC and integrin αv inhibitors (lanes 2 and 3) on the nuclear localization of RUNX2. Untreated (-) PC3 cells were used as control. Nuclear lysate proteins (N) were immunoblotted with a RUNX2 antibody. **B**-**D**: Loading control antibodies to GAPDH (C) and nucleoporin (B and D) were used to estimate relative amounts of total and nuclear proteins loaded in each lane. The results shown are representative of three independent experiments.

Cooperation between RUNX2 and Smads appears to be structurally coupled [24] and this seems to be important in eliciting biological signals that regulate the expression of osteoblast specific genes [26]. Therefore, we assessed in PC3 cells whether RUNX2 and Smad 5 were structurally linked. We used total cellular (T; 100 μg) and nuclear (N; 50 μg) lysates for immunoprecipitation with a RUNX2 antibody (Figure 7A). Immunoblotting was performed with a p-Smad 5 antibody. We show here co-precipitation of p-Smad 5 with RUNX2 in total cellular and nuclear lysates (A). However, the levels of immunoprecipitated p-Smad 5 (top panel) and co-immunoprecipitated RUNX2 (bottom panel) were higher in nuclear lysates (A, lane 2). As shown in Figure 5, RUNX2 present in the nucleus is phosphorylated on serine residues (Figure 7A; bottom panel). This suggests that the formation of a RUNX2-p-Smad 5 complex takes place in the nucleus and the complex is phosphorylated. 

Next we utilized RNA intereference to examine the effects of Smad5 knockdown in the nuclear localization of RUNX2. As shown in Figure 7B, Smad 5 level was reduced in a time-dependent manner at 48 h and 72 h (B-top panel, lanes 2 and 3) so did nuclear levels of RUNX2 (middle panel, lanes 2 and 3). These results indicate that RUNX2 nuclear localization of RUNX2 seems to be highly dependent on Smad 5 function.

### Alpha v beta 3-PKC dependent pathway regulates the phosphorylation of Smad 5

In an attempt to delineate the possible signaling pathway involved in the phosphorylation of Smad 5, PC3 cells were treated with a conventional PKC inhibitor (Gö6976; 100nM) and an inhibitor to αv (cyclic RGD; 100nM) for 16 h at 37^0^C as described previously [52]. Immunoblotting analysis of total cellular lysates (T, 100 μg) with an antibody to p-Smad 5 was performed. Our data show that these inhibitors blocked the phosphorylation of Smad 5 to a significant level (Figure 7C, lanes 2 and 3). Untreated PC3 cells were used as controls (lane 1). These data provides evidence that αvβ3 signaling regulates the phosphorylation of Smad 5, including PKC as an important signaling molecule within the αvβ3 signaling pathway.

We next asked whether inhibition of Smad 5 phosphorylation reduces the localization of RUNX2 in the nuclei (Figure 7D). We examined RUNX2 levels in the nuclear lysates (N, 50μg) made from PC3 cells treated with a αv and PKC inhibitor (lanes 2 and 3). A decrease in the levels of RUNX2 in cells treated with inhibitors (lanes 2 and 3) corresponds with the decrease in the phosphorylation of Smad 5 (Figure 7C, lanes 2 and 3). Following these interesting and novel findings, we suggest that phosphorylation of Smad 5 is an indispensable step for RUNX2 function.

### Alpha v beta 3-dependent pathway regulates the expression of RANKL

**Figure 8:**
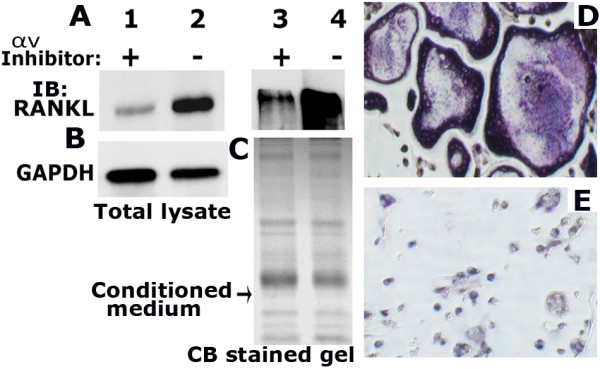
**The effect of integrin αv inhibitor on RANKL expression and osteoclast differentiation. A**-**C**: Western blot analysis. Equal amount of total cellular lysates (50 μg protein; A, lanes 1 and 2) and conditioned media (CM-20 μg protein; lanes 3 and 4) were immunoblotted with a RANKL antibody. CM was used to detect the secreted levels of RANKL. The blot in A was reprobed with a GAPDH antibody after stripping (B). GAPDH level was used as a control for loading. The loading control for the CM is shown by the use of Coomassie blue staining of a gel ran in parallel (C). **D** and **E**. The effect of CM on osteoclast differentiation in vitro is shown. TRAP-positive osteoclasts are stained in dark purple. Images were captured (X 200) with an inverted phase contrast microscope. The results shown are representative of three independent experiments.

We next examined whether inhibition of αv signaling reduces RANKL levels in PC3 cells (Figure 8A) and osteoclast differentiation in vitro (D and E). A decrease in the cellular (Figure 8A, lane 1) and secreted (lane 3) levels of RANKL was observed in PC3 cells treated with an inhibitor to αv (indicated +; Figure 8). Conditioned media from PC3 cells treated with a αv inhibitor failed to support differentiation of mouse bone marrow cells into multinucleated osteoclasts in vitro (Figure 8E). Multinucleated giant osteoclasts were observed in bone marrow cultures treated with CM media from control PC3 cells (Figure 8D). Taken together, our results indicate that the formation of the nuclear RUNX2/p-Smad 5 complex is a critical mechanism within metastatic prostate cancer cells to facilitate the expression of RANKL.

### Phosphorylation of RUNX2 and Smad 5 as well as RANKL expression are significantly increased in human prostate cancer

**Figure 9:**
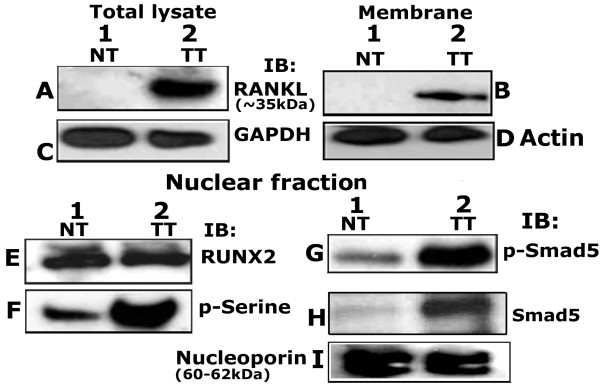
**Western analyses in prostatic normal and tumor lysates.** Total cellular (**A** and **C**), membrane (**B** and **D**) and nuclear (**E** to **I**) lysates from normal (NT) and prostatic tumor (TT) tissue (~20 μg protein) were immunoblotted (IB) with a RANKL (A and B), RUNX2 (E), phosphoserine (p-Serine; F), phospho-Smad 5 (p-Smad 5; G) and Smad 5 (H) antibody. Equal loading of the protein was shown in total cellular, membrane and nuclear lysates by relevant immunoblotting analysis with antibodies to GAPDH (C), actin (D) and nucleoporin (I). The results shown are representative of three independent experiments with three different lysates purchased from the vendor.

Immunoblotting analyses of total cellular and membrane protein isolated from human tissues derived from normal prostate (NT) and prostate tumor (TT) were performed with an antibody to RANKL (Figure 9). Expression of RANKL was observed in the total cellular and membrane fractions of the lysate protein from TT (Figure 9A and B, lane 2). RANKL protein was below the level of detection in normal tissue lysates (Figure 9A and B, lane 1).

Next, immunoblotting analyses were performed in the nuclear fractions of NT and TT with antibodies to RUNX2, p-Serine, p-Smad 5 and Smad 5 proteins (Figure 9). While the protein levels remain the same in NT and TT (Figure 9E, lanes 1 and 2), phosphorylation of RUNX2 was markedly increased in the nuclear fraction of TT than NT (Figure 9F, lanes 1 and 2). On the other hand, levels of Smad 5 and p-Smad 5 were elevated in the nuclear fraction of prostatic TT lysates (Figure 9G and H; lane 2) as compared with NT (G and H, lane 1).

### RANKL expression is markedly elevated in human prostatic adenocarcinoma tissues

**Figure 10:**
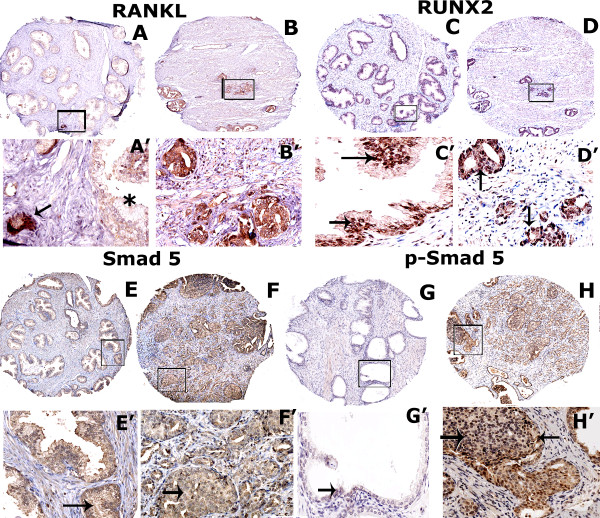
**Immunohistochemistry on TMA derived from normal and cancerous prostate tissue.** Immunohistochemical staining was performed in prostate cancer and normal tissue microarray with an antibody to RANKL (**A** and **B**), RUNX2 (**C** and **D**), Smad 5 (**E** and **F**) and p-Smad 5 (**G**-**H**). Normal tissue adjacent to prostate cancer are shown in A, C, E and G. Prostate carcinoma at grade 2–3 are shown in B, D, F and H. Arrows in **C**’, **D’G’** and **H’** point to nuclear localization of RUNX2 and p-Smad-5 proteins. An arrow in A’ points to a prostate carcinoma filled lumen (**A’**) adjacent to normal tissue (indicated by an asterisk; A’). Sections were immunostained (brown) with indicated primary antibody as described in the Methods section. Immunostained sections were counterstained with hematoxylin stain (blue). Magnification is 50X in A-H. Location of the high magnification (X200) regions shown in A’-H’ is indicated by a rectangle field in A-H. Staining was repeated two times.

To further validate the immunoblotting findings, we carried out immunohistochemistry analyses with antibodies to RANKL, RUNX2, Smad 5 and p-Smad 5 in a human prostate cancer tissue microarray (TMA). The specific tissue microarray used in this study contained 6 cases of prostatic adenocarcinoma with 6 adjacent normal tissues. Relative distribution of indicated proteins in immunostained TMA sections were semi-quantitatively analyzed by two other investigators and provided in Table 1. Sections shown in A, C, E and G have normal, hyperplastic and mildly dysplastic prostate tissue. Sections in B, D, F and H contain either moderately or poorly differentiated prostatic adenocarcinoma at grade 2 and 3. Hyperplastic, moderately differentiated prostatic tumor tissue contains luminal or basal epithelial cells. Moderately differentiated prostatic adenocarcinoma cells filling luminal space are indicted by arrows in the sections containing normal and hyperplastic prostate tissue (Figure 10, sections A’ and C’). High magnification regions shown below each of the cores is indicated by a corresponding rectangular field in top panels (A-H). Immunohistochemistry analyses (Figure 10) confirmed the observations shown in Figure 9 in the following aspects: a) RANKL expression increases in prostate cancer tissue (Figure 10B) as compared with normal tissue (Figure 10A). RANKL expression is higher in prostatic cancer tissue (indicated by an arrow in A’) adjacent to normal tissue (indicated by an asterisk in A’); b) Diffuse cytoplasmic and intense nuclear distribution of RUNX2 was observed in both normal and prostate cancer tissue sections (C, D, C’ and D’). The unavailability of the phospho-RUNX2 antibody prevented us from determining its localization in the normal and tumor prostatic tissue. However, based on immunoblotting analyses in PC3 nuclear lysates and human prostate cancer cells, we propose that RUNX2 localized in the nucleus of cancer tissue is mostly phosphorylated (Figure 7F, lane 2); c) Diffuse distribution of Smad-5 was observed in normal and prostate carcinoma sections. Distribution of Smad 5 is elevated in carcinoma tissues (F) as compared with normal tissue sections (E). Smad 5 staining was mostly cytoplasmic (E, F, E’ and F’). Phospho-Smad 5 (p-Smad 5) staining is very sparse in normal prostatic epithelial cells (G and G’) but predominates in sections containing adenocarcinoma cells (H and H’). Localization of p-Smad 5 was observed in the nuclei (indicated by arrows in G’ and H’).

**Table 1:** Expression of RANKL, RUNX2, Smad 5 and p-Smad 5 in prostatic carcinoma and normal tissue microarray sections

**Grade**	**Cells**	**RUNX2**	**Smad 5**	**p-Smad 5**	**RANKL**
Normal prostatic epithelial cells and PCa adjacent to these cells (n = 26)	Cancer cells appear normal	Normal cells = 63.0 ± 8%	Normal cells = 18.3 ± 6%	Normal cells = <6%	Normal cells = 22 ± 6.5%
		PCa = 28%	PCa = 33%	PCa = 16 ± 6%	PCa =12 ± 3.1%
		Stromal cells <5%	Stromal cells 8-10%		Stromal cells = <6%
Adenocarcinoma Grade 1 (n = 8) (Type: Malignant)	Cells appear normal and well differentiated	PCa = 60.7 ± 23%	PCa = 56.4 ± 8%**	PCa = 32 ± 5%**	PCa = 42 ± 8.4%**
		Stromal cells = <5%	Stromal cells =8%	Stromal cells = <5%	Stromal cells =12 ± 2.82%
Grade 2 (n = 12) (Type: Malignant)	Cells appear slightly different than normal	PCa = 71.3 ± 20%	PCa >63.3 ± 12%**	PCa >59 ± 14%**	PCa >46 ± 6.2%**
		Stromal cells ~5-8%	Stromal cells ~5%	Stromal cells ~5%	Stromal cells =10 ± 3.2%
Adenocarcinoma Grade 2–3 and 3 (n = 16) (Type:Malignant)	Cells appear abnormal Stroma is less.	PCa = 76 ± 8%	PCa >72 ± 11%**	PCa >78 ± 18%***	PCa = 65 ± 13%**

Prostatic carcinoma and normal tissue microarray containing 12 cases/24 cores was used. Stainings were repeated two times. Immunohistochemistry was performed with antibody to RANKL, RUNX2, Smad 5 and phospho-Smad 5 (p-Smad5). **p <0.01 and ***p <0.001 staining intensity vs. normal cells.

## Discussion

Expression of CD44 (standard or variant isoforms) has been considered a prognostic marker for the progression of prostate cancer. The mechanism by which CD44 regulates the progression of prostate cancer is largely unknown. The present study was performed to evaluate the role of CD44 in prostate cancer-induced bone metastasis. We screened three cell lines (PC3, DU145, and LNCaP) for the expression of CD44. Normal prostatic epithelial (HPR-1) and benign prostatic hyperplasic cells (BPH) were used as controls. PC3 and DU145 cells were established from the bone and brain metastatic lesions of a prostate cancer patient, respectively. Our studies are in agreement with the majority of earlier studies [53,54] in the expression of CD44 in androgen independent PC3 and DU145 cells, but not in androgen dependent LNCaP cells, which is established from a lymph node metastasis. Stable expression of androgen receptor in PC3 cells reduces CD44 expression to a significant level (data not shown).

The present study was undertaken to determine the possible mechanisms involved in the formation of osteolytic lesions associated with metastasis of prostate cancer cells to bone and the significance of CD44 and αvβ3 signaling. Previous studies in CD44 knockout mice link CD44 receptor with RANKL expression [47]. Our results in PC3 cells show that RANKL expression is in part mediated by CD44 signaling through RUNX2. As a result of CD44 expression, we have found expression of RANKL and MMP9 through RUNX2-dependent signaling in PC3 cells. RUNX2 SiRNA reduces MMP9 expression but not MMP2 at mRNA level. On the other hand, androgen-dependent LNCaP cells demonstrated expression and secretion of MMP2 as a major metalloproteases (Additional file 1: Figure S1). MMP2 expression may occur independent of RUNX2 and CD44 signaling in LNCaP cells. Consistent with our studies, others have shown negligible Runx2 in normal prostate epithelial and non-metastatic LNCaP cells. High Runx2 levels are associated with development of large tumors, increased expression of metastasis-related genes (MMP9, MMP13, VEGF, osteopontin) and secreted bone-resorbing factors (PTHrP, IL8) promoting osteolytic disease. Moreover, it was identified in co-culture studies that PC3 cells promote osteoclastogenesis and RUNX2 has a role in it [18]. This suggests a role for RUNX2 in the expression of RANKL.

RUNX proteins are expressed in prostate tissue and prostate cancer cells [18,55,56]. Breast and prostate cancers over expressing RUNX2 metastasized predominantly to bone [16,20]. We have shown a direct relationship of CD44 expression with RUNX2 activation in androgen-independent PC3 cells. Knockdown of CD44 reduced the expression of RUNX2 at mRNA and protein levels and hence reduced RUNX2-mediated signaling. Our studies demonstrate the possible role of CD44 signaling in RUNX2-mediated expression of RANKL. One possible explanation for RUNX2-regulated RANKL expression in PC3 cells may be associated with the lack of androgen receptor signaling. Androgen receptor was shown to bind RUNX2 and abrogates its binding to DNA and possibly to other nuclear DNAs [14]. It appears that CD44 expression in androgen-independent cells (e.g. PC3 cells) counteracts androgen receptor effects in terms of activation of RUNX2- mediated events. Therefore, knockdown of CD44 signaling in PC3 cells has the potential to reduce RUNX2 mediated signaling.

Hyaluronan (HA), the major non-protein glycosaminoglycan component of the extracellular matrix in mammalian bone marrow, functions in part through its receptor, CD44, to stimulate a series of intracellular signaling events that lead to RANKL expression [47]. We have shown previously that osteopontin (OPN) is secreted by PC3 cells. Over-expression of OPN in PC3 cells increases the secretion of RANKL through αvβ3 signaling [28]. Our current mechanistic evaluation studies in PC3 cells suggest a role for CD44 signaling in the phosphorylation of a RUNX2 and integrin αvβ3 signaling in the phosphorylation of Smad-5 independent of CD44 signaling. However, further studies are required to understand the precise contribution of downstream kinase(s) to the regulation of RUNX2 phosphorylation.

Runx2 nuclear localization was found to be up-regulated in prostate cancer and was suggested that this could be used as a predictor of metastasis in prostate cancer [57]. Several studies have shown that RUNX2 regulates localization of activated Smads in the subnuclear loci [24,58,59]. RUNX2 cooperates with Smads to induce differentiation of osteoblasts [26,60] and expression of collagenase in breast cancer cells [61]. RUNX2 forms complexes with Smad proteins as a requirement for mediating BMP/TGF β responsiveness in tumor cells. These effects contribute to tumor growth in bone and the accompanying bone loss in metastatic breast cancer cells [20]. Formation of the Runx2/Smad transcriptional complex is dependent on the phosphorylation state of these proteins [58]. Likewise, we detected predominant localization of phosphorylated RUNX2 and Smad 5 in the nuclei of lysates made from PC3 cells, prostatic adenocarcinoma and in tissue microarray sections containing primary prostatic tumor (grade 2–4).

Distinct relationship has been shown to exist between each Smad and RUNX2, [26,27,58,62,63]. Not only Smad 5 but also Smads 2 and 3 were shown to physically interact with RUNX2 in P19 embryonic carcinoma cells [23]. RUNX2/Smad 3 interaction stimulated collagen 3 expression in breast cancer cells [61]. Runx2/Smad3 complex negatively regulated endogenous and TGF-beta-induced connective tissue growth factor gene expression in vascular smooth muscle cells [64]. We have found that PC3 cells express Smad −2, -3 and −5 (Additional file 2: Figure S2). Smad 5 interaction was more with RUNX2 and this interaction regulates the expression of RANKL in prostate cancer cells.

RUNX2/Smad complex was shown to regulate the expression of RANKL in osteoblasts [24]. Although various studies have addressed the role of RUNX2 and Smad(s) in the regulation of expression of RANKL, the mechanisms underlying this process have remained largely unknown. Also the role of Smad5 in the expression of RANKL needs further elucidation. The data presented here show that Smad 5 and RUNX2 are co-immunoprecipitated in the nuclear fraction. RUNX2/Smad 5 complex regulates the expression of RANKL in PC3 cells. Interaction of RUNX2 with RANKL promoter was observed with CHIP assay. Binding of RUNX2 to the ctgg**aaccact**ggagt motif site on the RANKL is shown by CHIP assay. Although knockdown of RUNX2 or inhibition of phosphorylation of Smad-5 by an inhibitor to αv reduces the levels of RANKL, direct binding of Smad 5 with RANKL promoter was not observed. Future studies should delineate the relevant interactions between these proteins.

Interestingly, we have also observed reduced levels of RUNX2 and RANKL expression in cells treated with an inhibitor to αv or SiRNA to Smad5. These results indicate that RUNX2 is a major target gene of CD44 and Smad 5 signaling pathway. This is consistence with observations shown by others that Smad 5 is an upstream regulator of RUNX2 [26,51,60]. Over expression of Smad 5 increases RUNX2 levels in human MG63 osteosarcoma cells [51]. RUNX2 expression is transiently up regulated by TGF-β and BMP-2 activated Smads in mesenchymal precursor cell differentiation [26,60]. Smad 2 and 3 are expressed in PC3 cells; however, these proteins could not compensate the function of Smad 5. Therefore, it is possible that, a) Smad 5 which induces RUNX2 expression might also be translocated to subnuclear loci by RUNX2; b) Smad 2 or 3 interaction with RUNX2 may not occur for RANKL expression in response to integrin αvβ3 signaling. BMP2 signaling contributes to the high level of Runx2-Smad interaction which activates RANKL in osteoblasts. CD44/Smad signaling pathway has been shown to have a regulatory role in osteoblast differentiation in the absence of BMPs [65]. The underlying molecular mechanism by which αvβ3-activated Smad 5 regulates RUNX2 expression needs further elucidation. Taken together, bone metastatic prostate cancer cells (PC3) are osteomimetic and are expressing genes and proteins as observed in osteoblasts. However, the expression of osteoblastic specific genes in metastatic cancer cells does not necessarily involve the same pathway as observed in osteoblasts.

## Conclusions

Runx2 regulates early metastatic events in breast and prostate cancers, tumor growth, and osteolytic bone disease. Runx2 forms co-regulatory complexes with Smads in subnuclear domains to regulate gene transcription. Consideration is given to the potential for inhibition of this transcription factor as a therapeutic strategy upstream of the regulatory events contributing to the complexity of metastasis to bone. BMP/TGF-β and other growth factor signaling pathways regulate the formation of RUNX2/Smad complexes which in turn contribute to tumor growth in bone and the accompanying osteolytic disease (rev in [20]. As reported by others [58], we have also shown that RUNX2 and Smad 5 form a complex in the nuclei of PC3 cells and that both Smad5 and RUNX2 in nuclei are phosphorylated to form a transcriptional complex. Indeed, signaling by integrin αvβ3 and CD44 plays a role in the phosphorylation of Smad 5 and RUNX2, respectively. We have presented evidence that integration of two different signaling pathways (CD44 and αvβ3) facilitate osteoclastogenesis and bone loss via a RUNX2/Smad5/RANKL axis in metastatic prostate cancer cells. Crosstalk between integrin αvβ3 and CD44 signaling pathway assists in the phosphorylation of Smad 5 and RUNX2, respectively. Further study will be required for comprehensive understanding of the downstream signaling molecules involved in the phosphorylation of RUNX2 and Smad 5 and the details of sequence specific interaction between these proteins.

## Materials and methods

### Materials

Antibodies to RANKL, RUNX2, Histone and GAPDH as well as HRP-conjugated secondary antibodies (rabbit, goat and mice) were purchased from Santa Cruz Biotechnology, Inc. (Santa Cruz, CA). Antibodies to CD44 and sampler kit containing antibodies to Smads (phospho (P) -Smad1/5, P-Smad2, Smad2, Smad4, Smad 5 and Smad6) were purchased from Cell Signaling Technologies (Danvers, MA). Macrophage colony-stimulating factor-1 (MCSF-1) was purchased from R&D Systems (Minneapolis, MN). Cy2- and Cy3-conjugated secondary antibodies were purchased from Jackson Immunoresearch Laboratory, Inc. (West Grove, PA). An inhibitor to PKC (Gö6976) was purchased from Calbiochem (La Jolla, CA). A αv inhibitor (Cyclic RGD peptide) was purchased from Peptides International (Louisville, Kentucky). Complete mini protease inhibitor tablet was purchased from Roche Applied Science (Indianapolis, IN). Protein estimation reagent kit, molecular weight standards for proteins, and polyacrylamide solutions were purchased from Bio-Rad (Hercules, CA). Polyvinyldifluoride (PVDF) membrane for immunoblotting analysis and Amicon centrifugal concentrator devices for concentrating the protein in the conditioned media were obtained from Millipore Corp. (Bedford, MA). ECL reagent was purchased from Pierce (Rockford, IL). Vector Stain Elite and avidin-biotin complex (ABC) kit for immunohistochemistry were bought from Vector Laboratories (Burlingame, CA). Human prostate tumor and normal tissue lysates (total tissue, membrane and nuclear lysates) were purchased from Abcam (Cambridge, MA). TMAs containing 12 (24 cores) 24 (48 cores) and 40 (96 cores) cases were bought from US Biomax, Inc.

### Generation of PC3 cells knockdown of CD44

Four different silencing and one control scramble ShRNA constructs for the CD44 cDNA sequences (Genbank -NM_000610.3) were made using Shanghai Gene Pharm Corporation services (Shanghai, China). Target sequences for each of the silencing and scrambled ShRNA constructs are as follows:

1) 5^′^GCGCAGATCGATTTGAATATA-3^′^ (shCD44-492)

2) 5^′^GCTCCACCTGAAGAAG ATTGT-3^′^ (shCD44-801)

3) 5^′^-GCTTC ACCTACTGCAAATCC-3^′^ (shCD44-1874)

4) 5^′^-GGA AGAAGATAAAGACCATCC-3^′^ (shCD44-1994)

5) Scrambled ShRNA 5^′^-GCATGTAGCGTTCGTAAATAA-3′ (shCD44-scramble). Constructs were generated in pGPU6/GFP/Neo-vector. PC3 cells were transfected with these constructs and vector DNA using lipofectamine 2000 according to the manufacturer’s instructions (Invitrogen, Carlsbad, CA). Cells were cultured in Roswell Park Memorial Institute-1640 (RPMI 1640) media containing 10% FBS. After 24 h transfection, the cells were selected using G418 sulfate in the same medium. G418 sulfate resistant cells were analyzed for CD44 levels by immunoblotting with an antibody to CD44. The constructs which gave the best silencing effect of CD44 in PC3 cells were used for the isolation of individual clones. A significant decrease in the levels of CD44 was observed with shCD44-492 and −801 constructs. Individual clones (about 15–25) were isolated for each construct and cultured in complete medium containing G418 sulfate (200 μg/ml). About two to three clones from each construct (492 and 801) demonstrated a considerable decrease in the levels of CD44. Individual clones from each construct that exhibited highest levels of reduction in endogenous CD44 levels were used for the experiments described here. These cells were designated as PC3/Si (CD44).

### Cell culture

Prostate cancer cells (PC3, PC3 derived cell lines, LNCaP and DU145) and benign prostatic hyperplasic cells (BPH-1) were cultured in RPMI 1640 medium containing 5% or 10% fetal bovine serum (FBS) [28,66]. HPR-1 cells were cultured in keratinocyte medium supplemented with epidermal growth factor (EGF) (2.5 mg/500 ml) and bovine pituitary extracts (25 mg/500 ml) (Gibco BRL, Life Technologies, Bethesda, MD) as described previously [67]. Media were supplemented with penicillin and streptomycin (1%) and the cells were maintained at 37°C in a humidified incubator with 5% CO_2_.

### Quantification of RANKL in the conditioned medium

Cells of interest were grown to 80-90% confluence in RPMI-1640 medium containing 10% FBS. Cultures were then switched to serum-free RPMI-1640 medium for 72 h. The harvested CM was concentrated with Amicon centrifugal filter devices (Millipore Corporation, Bedford, MA). Protein concentrations were measured using the Bio-Rad protein assay reagent kit. Quantification of the secreted RANKL in the conditioned media was done by comparative analysis with different concentrations of either BSA or purified GST-RANKL using 12% polyacrylamide gel containing SDS (SDS-PAGE). Coomassie staining of the SDS-PAGE and immunoblotting with a RANKL antibody were performed to determine the concentration of RANKL in the medium [28].

### Preparation of osteoclast precursors

Mouse osteoclasts were generated in vitro using mouse bone marrow cells as described previously [68]. Cells isolated from five mice were cultured into 100-mm dishes with 20 ml of α-MEM medium supplemented with 10% fetal bovine serum (α-10). After culturing for 24 h, non-adhered cells were layered on histopaque-1077 (Sigma) and centrifuged at 300 × g for 15 min at room temperature. The cell layer between the histopaque and the media was removed and washed with α–10 medium at 2000 rpm for 7 min at room temperature. Cells were resuspended in α-10 media and cultured with the appropriate concentrations of M-CSF-1 (10 ng/ml) and RANKL (55–75 ng/ml). In order to determine the effect of secreted RANKL on osteoclast differentiation, mouse bone marrow cells were treated in the same way with M-CSF-1 but with conditioned medium (CM; 50-100 μg protein). CM collected from PC3, PC3-derived cell lines, DU145, LNCaP, BPH, and HPR-1 were used for osteoclast differentiation. After 3 days in culture, cultures were added with fresh α–10 medium containing M-CSF1 and respective CM. Multinucleated osteoclasts were observed from day 4 onwards. About 75-80% TRAP-positive multinucleated giant osteoclasts were observed from day 5 onwards [69].

### Treatment of PC3 cells with SiRNA to Smad 5 and inhibitors and preparation of total cellular lysates

PC3 cells cultured in RPMI-1640 media containing 10% FBS at 37°C were treated with PKC inhibitor (Go6976; 100nM) or integrin αv inhibitor (cyclic RGD; 100nM) for 16 h. SiRNA and non-targeting SiRNA control nucleotides for Smad 5 were purchased from Santa Cruz biotechnology, Inc. (Catalog No. sc-38378). Transfection was performed with lipofectamine as described previously [70]. Scrambled and SiRNA nucleotides were used to a final concentration of 50 nM for 48 and 72 h. Following various treatments, cells were washed three times with cold PBS and added with cold RIPA lysis buffer (10 mM Tris–HCl, pH 7.2, 150 mM NaCl, 1% deoxycholate, 1% Triton X-100, and 0.1% SDS) [71]. Lysis buffer was supplemented with EDTA- free complete mini protease inhibitor cocktail (1 tablet per 10 ml lysis buffer) immediately before use. After incubating on ice for 10 min, lysates were centrifuged for 5 min at 6,000 rpm at 4°C. The supernatants were saved and protein concentrations were measured using the Bio-Rad protein assay reagent kit. Protein lysates were subjected to SDS-PAGE (8 or 12% gels) and Western blot analysis as described previously [71].

### Preparation of cytoplasmic and nuclear protein fractions

Cells were lysed in a lysis buffer containing 10 mM Tris pH 7.9, 1.5 mM MgCl2, 10 mM KCl, 0.5 mM EGTA and protease inhibitor (1 tablet/10 ml buffer). Lysate was centrifuged at 500 × g to separate the nuclear pellet from the supernatant. The supernatant was considered as a cytosolic fraction. The nuclear pellet was resuspended by pipetting up and down with a P200 pipette tip in a buffer containing 20 mM Tris pH 7.5, 25% glycerol, 1.5 mM MgCl_2_, 400 mM NaCl and 0.5 mM EGTA. The suspension was centrifuged at 20,000 × g for 15 min at 4°C and the supernatant was used as nuclear fraction. Equal concentration of lysate proteins were used for Western blot analysis [71].

### Immunostaining

PC3 cells were cultured on cover slips in a 30 mm dish for overnight at 37^0^C prior to staining. Cells were washed three times with PBS and fixed in 4% paraformaldehyde–PBS for 20 min. After washing three times with PBS, cells were permeablized with 0.1% Triton X–PBS for 15 min. Subsequently, cells were blocked and immunostained with antibodies (1:100 dilution) of interest as described previously [70]. Cells were then washed and counterstained with respective isotype specific IgG conjugated with CY2 and CY3 fluorophore for 2-3 h at 4^0^C. The cells were washed and mounted on a slide in a mounting solution (Vector Laboratories, Inc.). The immunostained cells were viewed and photographed on a Bio-Rad confocal laser-scanning microscope. Images were stored in TIF image format and processed by the Adobe Photoshop software program (Adobe Systems, Inc., Mountain View, CA).

### RNA extraction and quantitative real-time PCR with RUNX2

Total RNA from different cell lines was isolated with TRIzol kit protocol with the DNA digest (Invitrogen, Carlsbad, CA). Reverse transcription reaction was performed in a 20 μl-reaction volume with 1 μg of total RNA by following the instructions provided by the manufacturer (Invitrogen, Carlsbad, CA). The cDNA was stored at -20^0^C until further use. For real time PCR, Runx2 primers (forward-5^′^CGGCCCTCCCTGAACTCT3^′^; reverse- 5^′^TGCCTGCCTGGGGTCTGTA3^′^) were used [55]. Glyceraldehyde-3-phosphate dehydrogenase (GAPDH) (forward-5^′^ TGCA CCACCAACTGCTTAG3^′^ and reverse-5^′^GATGCAGGGATGATGTTC3^′^) was used for normalization. Each reaction was performed in duplicates or triplicates in 25 μl volume in 96-well plates with a SYBR green reaction mix (Applied Biosystems Group) in an ABI 7000HT thermocycler (2 min at 50°C, 10 min at 95°C and 40 cycles of 15 s at 94°C and 1 min at 60°C) with 600-900nM primers as described previously [72]. The expression was calculated relative to that of control cells and normalized for GAPDH measured under the same conditions (Applied Biosystems/Roche, Branchburg, NJ), using the 2–ΔΔCT method [73].

### Immunohistochemistry

Prostatic adenocarcinoma tissue microarray (TMA) sections containing 6 cases of prostate adenocarcinoma with 6 adjacent normal prostate tissues in duplicate cores per case *were purchased from the US Biomax, Inc (*Rockville, MD)*.* TMA sections were processed, stained, and analyzed essentially as described previously [74]*.* Antigen retrieval was done using a buffer containing 10 mM Tris base pH 9, 1 mM EDTA and 0.05%Tween 20 in a microwave for 20 min. After incubation with 3% hydrogen peroxide in PBS for 30 min., sections were washed with PBS and then blocked either in 2.5% BSA or horse serum in PBS for 1 h at RT. Sections were then incubated with the primary antibodies diluted in blocking solution overnight at 4°C. After washing with PBS, slides were incubated with biotinylated secondary antibodies (1:400 dilutions) for 1 h, followed by the avidin-biotin complex (ABC) method using ABC kit (Vector Laboratories, Burlingame, CA) for 30 min. Slides were washed and developed in 3,3-diaminobenzidine (DAB) for 2–3 min. Immunostained sections were counterstained with hematoxylin, dehydrated and mounted with Permount (Fisher Scientific). Immunostained sections were scanned using an Aperio Scanscope® CS instrument (Aperio scanscope CS system, Vista, CA). Relative distribution of interested proteins in immunostained TMA sections were semi-quantitatively analyzed by two other investigators as well.

### Reverse transcription- polymerase chain reaction *(*RT-PCR) analysis

RT-PCR was done as described previously [70]. *Total RNA* was isolated and cDNAs were synthesized using 2 μg of total RNA. RT-PCR was done with the following primers: RUNX2 (406-bp product) - forward, 5^′^ ATTTAGGGCGCATTCCTCATC-3^′^ and reverse, 5^′^-TGTAATC TGACTCTGTCCTTGTGGAT-3^′^. GAPDH level was used for normalization. Samples were electrophoresed on an agarose gel and stained with ethidium bromide.

Chromatin immunoprecipitation assay (ChIP) was performed according to the manufacturer’s guidelines (Millipore, Cat#-17-295) and as described previously [75]. Briefly, PC3 cells were fixed by adding formaldehyde (Sigma, St. Louis, MO) to the medium to a final concentration of 1%. After 15 min the cells were washed, resuspended in CHIP-lysis buffer (Millipore) and sonicated. Immunoprecipitation was carried out at 4^0^C overnight using anti-RUNX2 (2 μg; rabbit polyclonal antibody) or non-immune rabbit IgG as a control. Immune complexes were washed, eluted and protein-DNA cross linking was reversed according to the manufacturer’s protocol. Immunoprecipitated DNA was quantified by RT-PCR using primer pairs (forward-5^′^ CTGCGTCTTCTTTAACCCATCT3^′^; reverse- 5^′^CCCTCCCTCTCTCTCAAT CTCT3^′^) in the RANKL promoter with expected product size 153 bp.

### Statistical analysis

All experiments were performed in triplicates and repeated three to four times and values presented as mean ± SEM. A value of p <0.05 was considered significant. Statistical significance was determined by analysis of variance (ANOVA) with the Bonferonni corrections (Instat for IBM; Graph pad software; San Diego, CA).

## Abbreviations

PKC: Protein kinase C; TMA: Tissue microarray; RANKL: Receptor activator of NFκb ligand; SMID: Smad interacting domain; Chip: Chromatin immunoprecipitation, PCR, Polymerase chain reaction, RT-PCR, Reverse transcriptase PCR, TMA, Tissue microarray; IP: Immunoprecipitation; IB: Immunoblot; CM: Conditioned medium; RUNX2: Runt-related transcription factor 2; SMAD: The gene products of the C. elegans gene **S**ma and the Drosophila gene ‘Mothers Against Decapentaplegic’ (Mad). SMAD proteins are signal transducers and transcriptional modulators; p-Smad 5: Phosphorylated Smad 5; PKC: Protein Kinase C; Integrin αvβ3: Vitronectin receptor; CD44: Cluster of Differentiation 44 (also known as cell surface adhesion receptor); SiRNA: Small interfering RNA; ShRNA: Short hairpin RNA; MMP: Matrix metalloproteinase; M-CSF: Macrophage colony stimulating factor.

## Competing interests

The authors declare that they have no competing interests.

## Authors’ contributions

AG carried out major experiments including Western blotting with human normal and tumor tissue lysates, immunohistochemistry on TMA, analyses with conditioned medium (Western blotting and osteoclast differentiation), studies with inhibitors (αv and PKC) and SiRNA (Smad 5). AG also participated in the MS preparation, statistical analysis of the data, discussion and interpretation of results. WC generated CD44 knockdown stable PC3 cell lines. MAC conceived the study, confocal microscopy analysis of immunostained PC3 cells, RUNX2 knockdown experiments and manuscript preparation. All authors read and approved the final manuscript.

## Grant support

This work was supported by National Institute of Health (NIH) grants AR46292 to MAC and training grant T32 DE007309 to AG. Wei Cao was supported in part by the National Natural Science Foundation of China (grant 30973343) and projects of the Shanghai Science and Technology Committee (grant 10XD1402500).

## Supplementary Material

Additional file 1 **Figure S1.**Analysis of the effects of SiRNA to RUNX2 on MMP9 and MMP2 RNA and protein levels (A-E) and revelation of major MMPs present in PC3 and LNCaP cells (F). **A-D**: We determined the effects of RUNX2 knockdown on the expression of MMP9 and MMP2 at mRNA (Figure S1-A) and protein levels (Figure S1D) in PC3 cells. Dose-dependent decrease in the levels of RUNX2 expression was observed in PC3 cells treated with SiRNA to RUNX2 at concentrations of 10, 20, and 50nM. The decrease was maximal (>90%) at 50nM RUNX2 SiRNA (A, lane 4). PC3 cells treated with scrambled RNAi (50nM) were used as control (A, lane 1). SiRNA to RUNX2 had very negligible effects on the changes in the levels of mRNA expression of MMP2 in PC3 cells (lane 6). GAPDH was used as internal control (Figure S1-B). A decrease in the expression of MMP9 at mRNA (Figure S1-A, lane 4) parallels with the MMP9 activity (~ 90kDa) in the conditioned medium isolated from cultures of PC3 cells treated with RUNX2 SiRNA (Figure S1-E, lane 3). MMP9 activity was determined by zymogram analysis. About 50μg membrane protein was used for the gelatin zymography to determine the activities of MMP9 (S1-E). As shown previously [Ref.28], only the active form of MMP-9 was observed in the conditioned medium (Figure S1-E, lanes 1-3). The activity of a recombinant MMP-9 protein containing pro- and active band was used as an identification marker (lane 4 in S1-E). Furthermore, the decrease in the protein levels of RUNX2 (~55kDa) in SiRNA to RUNX2 treated cells (Figure S1-C, lane 3) corresponds with a decrease in the total cellular protein levels of MMP 9 (Figure S1-D, lane 3) but not MMP 2 (~72kDa). MMP 2 levels remain the same in control untreated as well as scrambled RNAi and SiRNA to RUNX2 treated cells (Figure S1- D). These results imply that the RUNX2 is not a direct binding factor to induce transcriptional activation of MMP 2.
**F**: Zymogram analysis with normal prostatic epithelial cells (HPR1) was used as a control (lane 4) for prostate cancer cells derived from lymph node (LNCaP, lane 2) and bone (PC3, lane 3) metastases. The activity of a recombinant MMP2 and MMP9 protein containing pro and active bands (indicated by arrows) were used as an identification marker (lane 1). LNCaP cells demonstrated MMP2 as a major metalloproteases where as MMP9 was observed as major MMP although MMP2 was observed at mRNA (Figure 1A) and protein levels (Figure S1-D and F) in PC3 cells. About 75μg total cellular protein was used for zymogram analysis as shown previously [ref. [28].
**Method**: Gelatin zymography: Conditioned media collected from various PC3 cell lines were concentrated approximately 10-fold) with a centricon concentrator (Amicon, Beverly, MA). Ten micrograms of concentrated media protein in 10-20 μl were mixed with gel loading buffer with no reducing agent (βME or DTT) and incubated at RT for 10-15 min. SDS-PAGE containing 0.1% gelatin was used for electrophoresis. Samples were loaded without heating with sample buffer. After electrophoresis, gels were incubated overnight in a buffer containing 50 mM Tris-HCl, pH 7.6, 5 mM CaCl_2_, 1 μM ZnCl_2_, and 1% Triton X-100. Triton was used to remove SDS from the gel. Gels were then stained with Coomassie brilliant blue for 2-3 h and destained with 7% acetic acid or water. Gelatinolytic activity was detected as clear bands in the background of blue staining [ref. [28]. Click here for file

Additional file 2 **Figure S2.**Immunoblotting analysis for Smad 2, 3, 5 and 6 proteins in PC3 cells.
About 50μg total cellular lysate protein was used for immunoblotting with antibodies to phospho-Smad (p-Smad) -2 (60kDa; lane 1), -3 (52 kDa; lane 2), -5 (60kDa; lane 3) and -6 (62kDa; lane 4). Blots were reprobed with an antibody to GAPDH after stripping. Phosphorylation of 2, 3, and 5 was observed in PC3 cells. However, Smad- 5 phosphorylation is significantly more than Smad-2 and 3 (lanes 1 and 2). Phosphorylation of Smad-6 is really negligible or not observed. Click here for file

## References

[B1] van der Gulden JW Kiemeney LA Verbeek AL Straatman H Mortality trend from prostate cancer in The Netherlands (1950–1989) 7 Prostate 1994 24 33 38 10.1002/pros.2990240108 8290387

[B2] Brawley OW Prostate cancer epidemiology in the United States World J Urol 2012 30 195 200 10.1007/s00345-012-0824-2 22476558

[B3] Carlin BI Andriole GL The natural history, skeletal complications, and management of bone metastases in patients with prostate carcinoma 1 Cancer 2000 88 2989 2994 10.1002/1097-0142(20000615)88:12+<2989::AID-CNCR14>3.0.CO;2-Q 10898342

[B4] Sanchez-Sweatman OH Orr FW Singh G Human metastatic prostate PC3 cell lines degrade bone using matrix metalloproteinases Invasion Metastasis 1998 18 297 305 10.1159/000024522 10729774

[B5] Dougall WC RANKL signaling in bone physiology and cancer Curr Opin Support Palliat Care 2007 1 317 322 10.1097/SPC.0b013e3282f335be 18685382

[B6] Hofbauer LC Schoppet M Clinical implications of the osteoprotegerin/RANKL/RANK system for bone and vascular diseases12 JAMA 2004 292 490 495 10.1001/jama.292.4.490 15280347

[B7] Lacey DL Timms E Tan H-L Kelley MJ Dunstan CR Burgess T Osteoprotegerin ligand is a cytokine that regulates osteoclast differentiation and activation Cell 1998 93 165 176 10.1016/S0092-8674(00)81569-X 9568710

[B8] Zhang J Dai J Qi Y Lin DL Smith P Strayhorn C Osteoprotegerin inhibits prostate cancer-induced osteoclastogenesis and prevents prostate tumor growth in the bone J Clin Invest 2001 107 1235 1244 10.1172/JCI11685 11375413 PMC209296

[B9] Zhang J Dai J Yao Z Lu Y Dougall W Keller ET Soluble receptor activator of nuclear factor kappaB Fc diminishes prostate cancer progression in bone Cancer Res 2003 63 7883 7890 14633717

[B10] Miller RE Roudier M Jones J Armstrong A Canon J Dougall WC RANK ligand inhibition plus docetaxel improves survival and reduces tumor burden in a murine model of prostate cancer bone metastasis Mol Cancer Ther 2008 7 2160 2169 10.1158/1535-7163.MCT-08-0046 18606716

[B11] Yonou H Ochiai A Ashimine S Maeda H Horiguchi Y Yoshioka K The bisphosphonate YM529 inhibits osteoblastic bone tumor proliferation of prostate cancer2 Prostate 2007 67 999 1009 10.1002/pros.20592 17440967

[B12] Schneider A Kalikin LM Mattos AC Keller ET Allen MJ Pienta KJ Bone turnover mediates preferential localization of prostate cancer in the skeleton Endocrinology 2005 146 1727 1736 10.1210/en.2004-1211 15637291

[B13] Liao J McCauley LK Skeletal metastasis: Established and emerging roles of parathyroid hormone related protein (PTHrP) Cancer Metastasis Rev 2006 25 559 571 17165129 10.1007/s10555-006-9033-z

[B14] Baniwal SK Khalid O Sir D Buchanan G Coetzee GA Frenkel B Repression of Runx2 by androgen receptor (AR) in osteoblasts and prostate cancer cells: AR binds Runx2 and abrogates its recruitment to DNA Mol Endocrinol 2009 23 1203 1214 10.1210/me.2008-0470 19389811 PMC2718746

[B15] Barnes GL Hebert KE Kamal M Javed A Einhorn TA Lian JB Fidelity of Runx2 activity in breast cancer cells is required for the generation of metastases-associated osteolytic disease Cancer Res 2004 64 4506 4513 10.1158/0008-5472.CAN-03-3851 15231660

[B16] Brubaker KD Vessella RL Brown LG Corey E Prostate cancer expression of runt-domain transcription factor Runx2, a key regulator of osteoblast differentiation and function Prostate 2003 56 13 22 10.1002/pros.10233 12746842

[B17] Javed A Barnes GL Pratap J Antkowiak T Gerstenfeld LC van Wijnen AJ Impaired intranuclear trafficking of Runx2 (AML3/CBFA1) transcription factors in breast cancer cells inhibits osteolysis in vivo Proc Natl Acad Sci U S A 2005 102 1454 1459 10.1073/pnas.0409121102 15665096 PMC547873

[B18] Akech J Wixted JJ Bedard K van der Deen M Hussain S Guise TA Runx2 association with progression of prostate cancer in patients: mechanisms mediating bone osteolysis and osteoblastic metastatic lesions Oncogene 2010 29 811 821 10.1038/onc.2009.389 19915614 PMC2820596

[B19] Selvamurugan N Shimizu E Lee M Liu T Li H Partridge NC Identification and characterization of Runx2 phosphorylation sites involved in matrix metalloproteinase-13 promoter activation FEBS Lett 2009 583 1141 1146 10.1016/j.febslet.2009.02.040 19264160 PMC4640702

[B20] Pratap J Lian JB Javed A Barnes GL van Wijnen AJ Stein JL Regulatory roles of Runx2 in metastatic tumor and cancer cell interactions with bone Cancer Metastasis Rev 2006 25 589 600 17165130 10.1007/s10555-006-9032-0

[B21] Pratap J Wixted JJ Gaur T Zaidi SK Dobson J Gokul KD Runx2 transcriptional activation of Indian Hedgehog and a downstream bone metastatic pathway in breast cancer cells Cancer Res 2008 68 7795 7802 10.1158/0008-5472.CAN-08-1078 18829534 PMC2596479

[B22] Kitazawa R Mori K Yamaguchi A Kondo T Kitazawa S Modulation of mouse RANKL gene expression by Runx2 and vitamin D3 J Cell Biochem 2008 105 1289 1297 10.1002/jcb.21929 18814144

[B23] Hanai J Chen LF Kanno T Ohtani-Fujita N Kim WY Guo W-H Interacton and functional cooperation of PEBP2/CBF with Smads J Biol Chem 1999 274 31577 31582 10.1074/jbc.274.44.31577 10531362

[B24] Javed A Afzal F Bae JS Gutierrez S Zaidi K Pratap J Specific residues of RUNX2 are obligatory for formation of BMP2-induced RUNX2-SMAD complex to promote osteoblast differentiation Cells Tissues Organs 2009 189 133 137 10.1159/000151719 18728344 PMC2701265

[B25] Ito Y Zhang YW A RUNX2/PEBP2alphaA/CBFA1 mutation in cleidocranial dysplasia revealing the link between the gene and Smad J Bone Miner Metab 2001 19 188 194 10.1007/s007740170041 11368305

[B26] Lee KS Kim HJ Li QL Chi XZ Ueta C Komori T Runx2 is a common target of transforming growth factor beta1 and bone morphogenetic protein 2, and cooperation between Runx2 and Smad5 induces osteoblast-specific gene expression in the pluripotent mesenchymal precursor cell line C2C12 Mol Cell Biol 2000 20 8783 8792 10.1128/MCB.20.23.8783-8792.2000 11073979 PMC86511

[B27] Jun JH Yoon WJ Seo SB Woo KM Kim GS Ryoo HM BMP2-activated Erk/MAP kinase stabilizes Runx2 by increasing p300 levels and histone acetyltransferase activity J Biol Chem 2010 285 36410 36419 10.1074/jbc.M110.142307 20851880 PMC2978570

[B28] Desai B Rogers MJ Chellaiah MA Mechanisms of osteopontin and CD44 as metastatic principles in prostate cancer cells Mol Cancer 2007 6 18 10.1186/1476-4598-6-18 17343740 PMC1828067

[B29] Cooper CR Chay CH Pienta KJ The role of alpha(v)beta(3) in prostate cancer progression Neoplasia 2002 4 191 194 10.1038/sj.neo.7900224 11988838 PMC1531692

[B30] Weber GF Ashkar S Molecular mechanisms of tumor dissemination in primary and metastatic brain cancers Brain Res Bull 2000 53 421 424 10.1016/S0361-9230(00)00379-8 11136998

[B31] Pecheur I Peyruchaud O Serre CM Guglielmi J Voland C Bourre F Integrin alpha(v)beta3 expression confers on tumor cells a greater propensity to metastasize to bone FASEB J 2002 16 1266 1268 12153995 10.1096/fj.01-0911fje

[B32] Boucharaba A Serre CM Gres S Saulnier-Blache JS Bordet JC Guglielmi J Platelet-derived lysophosphatidic acid supports the progression of osteolytic bone metastases in breast cancer J Clin Invest 2004 114 1714 1725 15599396 10.1172/JCI22123 PMC535068

[B33] Naor D Sionov RV Zahalka M Rochman M Holzmann B Ish-Shalom D Organ-specific requirements for cell adhesion molecules during lymphoma cell dissemination Curr Top Microbiol Immunol 1998 231 143 166 10.1007/978-3-642-71987-5_9 9479865

[B34] Sy MS Guo YJ Stamenkovic I Distinct effects of two CD44 isoforms on tumor growth in vivo J Exp Med 1991 174 859 866 10.1084/jem.174.4.859 1919439 PMC2118964

[B35] Gao AC Lou W Dong JT Isaacs JT CD44 is a metastasis suppressor gene for prostatic cancer located on human chromosome 11p13 Cancer Res 1997 57 846 849 9041184

[B36] Noordzij MA Van Steenbrugge GJ Schroder FH Van Der Kwast TH Decreased expression of CD44 in metastatic prostate cancer Int J Cancer 1999 84 478 483 10.1002/(SICI)1097-0215(19991022)84:5<478::AID-IJC5>3.0.CO;2-N 10502724

[B37] Tanne Y Tanimoto K Tanaka N Ueki M Lin YY Ohkuma S Expression and activity of Runx2 mediated by hyaluronan during chondrocyte differentiation Arch Oral Biol 2008 53 478 487 10.1016/j.archoralbio.2007.12.007 18242579

[B38] Hayer S Steiner G Gortz B Reiter E Tohidast-Akrad M Amling M CD44 is a determinant of inflammatory bone loss J Exp Med 2005 201 903 914 10.1084/jem.20040852 15781582 PMC2213110

[B39] Cao J Singleton P Majumdar S Burghardt A Bourguignon GJ Halloran BP Hyaluronan increases RANKL expression in mouse primary osteoblasts through CD44. A potential role in age-related bone loss J Bone Miner Res 2003 18 S2 S78 Ref Type: Abstract

[B40] Ricciardelli C Russell DL Ween MP Mayne K Suwiwat S Byers S Formation of hyaluronan- and versican-rich pericellular matrix by prostate cancer cells promotes cell motility J Biol Chem 2007 282 10814 10825 10.1074/jbc.M606991200 17293599

[B41] Klarmann GJ Hurt EM Mathews LA Zhang X Duhagon MA Mistree T Invasive prostate cancer cells are tumor initiating cells that have a stem cell-like genomic signature Clin Exp Metastasis 2009 26 433 446 10.1007/s10585-009-9242-2 19221883 PMC2782741

[B42] Hurt EM Kawasaki BT Klarmann GJ Thomas SB Farrar WL CD44+ CD24(−) prostate cells are early cancer progenitor/stem cells that provide a model for patients with poor prognosis Br J Cancer 2008 98 756 765 10.1038/sj.bjc.6604242 18268494 PMC2259168

[B43] Dhir R Gau JT Krill D Bastacky S Bahnson RR Cooper DL CD44 Expression in Benign and Neoplastic Human Prostates Mol Diagn 1997 2 197 204 10.1016/S1084-8592(97)80029-X 10462610

[B44] Pratap J Javed A Languino LR van Wijnen AJ Stein JL Stein GS The Runx2 osteogenic transcription factor regulates matrix metalloproteinase 9 in bone metastatic cancer cells and controls cell invasion Mol Cell Biol 2005 25 8581 8591 10.1128/MCB.25.19.8581-8591.2005 16166639 PMC1265732

[B45] Grier DG Thompson A Kwasniewska A McGonigle GJ Halliday HL Lappin TR The pathophysiology of HOX genes and their role in cancer J Pathol 2005 205 154 171 10.1002/path.1710 15643670

[B46] Roccisana JL Kawanabe N Kajiya H Koide M Roodman GD Reddy SV Functional role for heat shock factors in the transcriptional regulation of human RANK ligand gene expression in stromal/osteoblast cells J Biol Chem 2004 279 10500 10507 14699143 10.1074/jbc.M303727200

[B47] Cao JJ Singleton PA Majumdar S Boudignon B Burghardt A Kurimoto P Hyaluronan increases RANKL expression in bone marrow stromal cells through CD44 J Bone Miner Res 2005 20 30 40 10.1359/JBMR.041014 15619667

[B48] Tai S Sun Y Squires JM Zhang H Oh WK Liang CZ PC3 is a cell line characteristic of prostatic small cell carcinoma Prostate 2011 71 1668 1679 10.1002/pros.21383 21432867 PMC3426349

[B49] Yu C Yao Z Dai J Zhang H Escara-Wilke J Zhang X ALDH activity indicates increased tumorigenic cells, but not cancer stem cells, in prostate cancer cell lines In Vivo 2011 25 69 76 21282737

[B50] Mori K Kitazawa R Kondo T Maeda S Yamaguchi A Kitazawa S Modulation of mouse RANKL gene expression by Runx2 and PKA pathway J Cell Biochem 2006 98 1629 1644 10.1002/jcb.20891 16598781

[B51] Chang SF Chang CA Lee DY Lee PL Yeh YM Yeh CR Tumor cell cycle arrest induced by shear stress: Roles of integrins and Smad Proc Natl Acad Sci U S A 2008 105 3927 3932 10.1073/pnas.0712353105 18310319 PMC2268796

[B52] Robertson BW Bonsal L Chellaiah MA Regulation of Erk1/2 activation by osteopontin in PC3 human prostate cancer cells Mol Cancer 2010 9 260 10.1186/1476-4598-9-260 20868520 PMC3098013

[B53] Yeung F Law WK Yeh CH Westendorf JJ Zhang Y Wang R Regulation of human osteocalcin promoter in hormone-independent human prostate cancer cells J Biol Chem 2002 277 2468 2476 10.1074/jbc.M105947200 11684680

[B54] Robertson BW Chellaiah MA Osteopontin induces beta-catenin signaling through activation of Akt in prostate cancer cells Exp Cell Res 2010 316 1 11 10.1016/j.yexcr.2009.10.012 19850036 PMC2787770

[B55] van der Deen M Akech J Wang T FitzGerald TJ Altieri DC Languino LR The cancer-related Runx2 protein enhances cell growth and responses to androgen and TGFbeta in prostate cancer cells J Cell Biochem 2010 109 828 837 20082326 10.1002/jcb.22463 PMC2925394

[B56] Fowler M Borazanci E McGhee L Pylant SW Williams BJ Glass J RUNX1 (AML-1) and RUNX2 (AML-3) cooperate with prostate-derived Ets factor to activate transcription from the PSA upstream regulatory region J Cell Biochem 2006 97 1 17 10.1002/jcb.20664 16237704

[B57] Chua CW Chiu YT Yuen HF Chan KW Man K Wang X Suppression of androgen-independent prostate cancer cell aggressiveness by FTY720: validating Runx2 as a potential antimetastatic drug screening platform Clin Cancer Res 2009 15 4322 4335 10.1158/1078-0432.CCR-08-3157 19509141

[B58] Afzal F Pratap J Ito K Ito Y Stein JL van Wijnen AJ Smad function and intranuclear targeting share a Runx2 motif required for osteogenic lineage induction and BMP2 responsive transcription J Cell Physiol 2005 204 63 72 10.1002/jcp.20258 15573378

[B59] Zaidi SK Sullivan AJ van Wijnen AJ Stein JL Stein GS Lian JB Integration of Runx and Smad regulatory signals at transcriptionally active subnuclear sites Proc Natl Acad Sci U S A 2002 99 8048 8053 10.1073/pnas.112664499 12060751 PMC123018

[B60] Lee KS Hong SH Bae SC Both the Smad and p38 MAPK pathways play a crucial role in Runx2 expression following induction by transforming growth factor-beta and bone morphogenetic protein Oncogene 2002 21 7156 7163 10.1038/sj.onc.1205937 12370805

[B61] Selvamurugan N Kwok S Partridge NC Smad3 interacts with JunB and Cbfa1/Runx2 for transforming growth factor-beta1-stimulated collagenase-3 expression in human breast cancer cells J Biol Chem 2004 279 27764 27773 10.1074/jbc.M312870200 15084595

[B62] Leboy P Grasso-Knight G D’Angelo M Volk SW Lian JV Drissi H Smad-Runx interactions during chondrocyte maturation J Bone Joint Surg Am 2001 83-A Suppl 1 S15 S22 11263661

[B63] Hjelmeland AB Schilling SH Guo X Quarles D Wang XF Loss of Smad3-mediated negative regulation of Runx2 activity leads to an alteration in cell fate determination Mol Cell Biol 2005 25 9460 9468 10.1128/MCB.25.21.9460-9468.2005 16227596 PMC1265845

[B64] Ohyama Y Tanaka T Shimizu T Matsui H Sato H Koitabashi N Runx2/Smad3 complex negatively regulates TGF-beta-induced connective tissue growth factor gene expression in vascular smooth muscle cells J Atheroscler Thromb 2012 19 23 35 10.5551/jat.9753 21986102

[B65] Tanikawa R Tanikawa T Hirashima M Yamauchi A Tanaka Y Galectin-9 induces osteoblast differentiation through the CD44/Smad signaling pathway Biochem Biophys Res Commun 2010 394 317 322 10.1016/j.bbrc.2010.02.175 20206131

[B66] Wang CY Lee LH Mutagenicity and antibacterial activity of hydroxamic acids Antimicrob Agents Chemother 1977 11 753 10.1128/AAC.11.4.753 856029 PMC352062

[B67] Choo CK Ling MT Chan KW Tsao SW Zheng Z Zhang D Immortalization of human prostate epithelial cells by HPV 16 E6/E7 open reading frames Prostate 1999 40 150 158 10.1002/(SICI)1097-0045(19990801)40:3<150::AID-PROS2>3.0.CO;2-7 10398276

[B68] Chellaiah M Kizer N Silva M Alvarez U Kwiatkowski D Hruska KA Gelsolin deficiency blocks podosome assembly and produces increased bone mass and strength J Cell Biol 2000 148 665 678 10.1083/jcb.148.4.665 10684249 PMC2169374

[B69] Gupta A Lee BS Khadeer MA Tang Z Chellaiah M Abu-Amer Y Leupaxin is a critical adaptor protein in the adhesion zone of the osteoclast J Bone Miner Res 2003 18 669 685 10.1359/jbmr.2003.18.4.669 12674328

[B70] Desai B Ma T Zhu J Chellaiah MA Characterization of the expression of variant and standard CD44 in prostate cancer cells: identification of the possible molecular mechanism of CD44/MMP9 complex formation on the cell surface J Cell Biochem 2009 108 272 284 10.1002/jcb.22248 19582779 PMC7198262

[B71] Chellaiah M Hruska KA Osteopontin stimulates gelsolin associated phosphoinositide levels and PtdIns 3-hydroxyl kinase Mol Biol Cell 1996 7 743 753 8744948 10.1091/mbc.7.5.743 PMC275927

[B72] Ma T Sadashivaiah K Chellaiah MA Regulation of sealing ring formation by L-plastin and cortactin in osteoclasts J Biol Chem 2010 285 29911 29924 10.1074/jbc.M109.099697 20650888 PMC2943304

[B73] Livak KJ Schmittgen TD Analysis of relative gene expression data using real-time quantitative PCR and the 2(−Delta Delta C(T)) Method Methods 2001 25 402 408 10.1006/meth.2001.1262 11846609

[B74] Schneider A Younis RH Gutkind JS Hypoxia-induced energy stress inhibits the mTOR pathway by activating an AMPK/REDD1 signaling axis in head and neck squamous cell carcinoma Neoplasia 2008 10 1295 1302 18953439 10.1593/neo.08586 PMC2570606

[B75] Singh IS Gupta A Nagarsekar A Cooper Z Manka C Hester L Heat shock co-activates interleukin-8 transcription Am J Respir Cell Mol Biol 2008 39 235 242 10.1165/rcmb.2007-0294OC 18367728 PMC2542457

[B76] Feng P Li TL Guan ZX Franklin RB Costello LC Direct effect of zinc on mitochondrial apoptogenesis in prostate cells Prostate 2002 52 311 318 10.1002/pros.10128 12210492 PMC4465826

