# Supplementary material for: Integrin αvβ3 and CD44 pathways in metastatic prostate cancer cells support osteoclastogenesis via a Runx2/Smad 5/receptor activator of NF-κB ligand signaling axis
Source: Mol Cancer. 2012 Sep 11;11:66. doi: 10.1186/1476-4598-11-66 (PMC3499378; doi:10.1186/1476-4598-11-66)
Supplement: Additional file 1 — Figure S1.Analysis of the effects of SiRNA to RUNX2 on MMP9 and MMP2 RNA and protein levels (A-E) and revelation of major MMPs present in PC3 and LNCaP cells (F). A-D: We determined the effects of RUNX2 knockdown on the expression of MMP9 and MMP2 at mRNA (Figure S1-A) and protein levels (Figure S1D) in PC3 cells. Dose-dependent decrease in the levels of RUNX2 expression was observed in PC3 cells treated with SiRNA to RUNX2 at concentrations of 10, 20, and 50nM. The decrease was maximal (>90%) at 50nM RUNX2 SiRNA (A, lane 4). PC3 cells treated with scrambled RNAi (50nM) were used as control (A, lane 1). SiRNA to RUNX2 had very negligible effects on the changes in the levels of mRNA expression of MMP2 in PC3 cells (lane 6). GAPDH was used as internal control (Figure S1-B). A decrease in the expression of MMP9 at mRNA (Figure S1-A, lane 4) parallels with the MMP9 activity (~ 90kDa) in the conditioned medium isolated from cultures of PC3 cells treated with RUNX2 SiRNA (Figure S1-E, lane 3). MMP9 activity was determined by zymogram analysis. About 50μg membrane protein was used for the gelatin zymography to determine the activities of MMP9 (S1-E). As shown previously [Ref.28], only the active form of MMP-9 was observed in the conditioned medium (Figure S1-E, lanes 1-3). The activity of a recombinant MMP-9 protein containing pro- and active band was used as an identification marker (lane 4 in S1-E). Furthermore, the decrease in the protein levels of RUNX2 (~55kDa) in SiRNA to RUNX2 treated cells (Figure S1-C, lane 3) corresponds with a decrease in the total cellular protein levels of MMP 9 (Figure S1-D, lane 3) but not MMP 2 (~72kDa). MMP 2 levels remain the same in control untreated as well as scrambled RNAi and SiRNA to RUNX2 treated cells (Figure S1- D). These results imply that the RUNX2 is not a direct binding factor to induce transcriptional activation of MMP 2. F: Zymogram analysis with normal prostatic epithelial cells (HPR1) was used as a control (lane 4) for pr [file 1476-4598-11-66-S1.doc]

**Results:**

Additional Figure 1


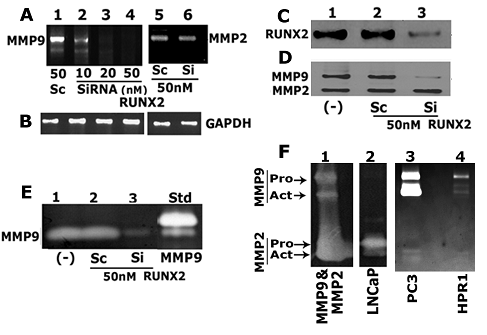


Additional Figure 2


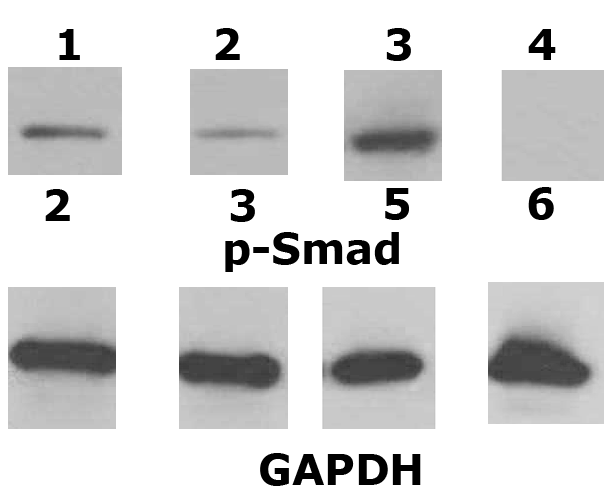


**Figure Legends**

Additional figure 1

We determined the effects of RUNX2 knockdown on the expression of MMP9 and MMP2 at mRNA (Ad-Figure 1A) and protein levels (Ad-Figure 1D) in PC3 cells. Dose-dependent decrease in the levels of RUNX2 expression was observed in PC3 cells treated with SiRNA to RUNX2 at concentrations of 10, 20, and 50nM. The decrease was maximal (>90%) at 50nM RUNX2 SiRNA (A, lane 4). PC3 cells treated with scrambled RNAi (50nM) were used as control (A, lane 1). SiRNA to RUNX2 had very negligible effects on the changes in the levels of mRNA expression of MMP2 in PC3 cells (lane 6). GAPDH was used as internal control (Figure 1B). A decrease in the expression of MMP9 at mRNA (Figure 1A, lane 4) parallels with the MMP9 activity (~ 90kDa) in the conditioned medium isolated from cultures of PC3 cells treated with RUNX2 SiRNA (Figure 1E, lane 3). MMP9 activity was determined by zymogram analysis. About 50µg membrane protein was used for the gelatin zymography to determine the activities of MMP9 (E). Consistent with our previous observations [Ref.28 in main document], only the active form of MMP-9 was observed in the conditioned medium (Figure 1E, lanes 1-3). The activity of a recombinant MMP-9 protein containing pro- and active band was used as an identification marker (lane 4 in E). Furthermore, the decrease in the protein levels of RUNX2 (~55kDa) in SiRNA to RUNX2 treated cells (Figure 1C, lane 3) corresponds with a decrease in the total cellular protein levels of MMP 9 (Figure 1D, lane 3) but not MMP 2 (~72kDa). MMP 2 levels remain the same in control untreated as well as scrambled RNAi and SiRNA to RUNX2 treated cells (Figure 1 D). These results imply that the RUNX2 is not a direct binding factor to induce transcriptional activation of MMP 2.

Figure 1F: Zymogram analysis with normal prostatic epithelial cells (HPR1) was used as a control (lane 4) for prostate cancer cells derived from lymph node (LNCaP, lane 2) and bone (PC3, lane 3) metastases. The activity of a recombinant MMP2 and MMP9 protein containing pro and active bands (indicated by arrows) were used as an identification marker (lane 1). LNCaP cells demonstrated MMP2 as a major metalloproteases where as MMP9 was observed as major MMP although MMP2 was observed at mRNA (Figure 1A) and protein levels (Figure 1D and F) in PC3 cells. About 75µg total cellular protein was used for zymogram analysis as shown previously [ref.28].

Additional figure 2

About 50µg total cellular lysate protein was used for immunoblotting with antibodies to phospho-Smad (p-Smad) -2 (60kDa; lane 1), -3 (52 kDa; lane 2), -5 (60kDa; lane 3) and -6 (62kDa; lane 4). Blots were reprobed with an antibody to GAPDH after stripping. Phosphorylation of 2, 3, and 5 was observed in PC3 cells. However, Smad- 5 phosphorylation is significantly more than Smad-2 and 3 (lanes 1 and 2). Phosphorylation of Smad-6 is really negligible or not observed.

**Method**

Gelatin zymography**:** Conditioned media collected from various PC3 cell lines were concentrated approximately 10-fold) with a centricon concentrator (Amicon, Beverly, MA). Ten micrograms of concentrated media protein in 10-20 μl were mixed with gel loading buffer with no reducing agent (βME or DTT) and incubated at RT for 10–15 min. SDS-PAGE containing 0.1% gelatin was used for electrophoresis. Samples were loaded without heating with sample buffer. After electrophoresis, gels were incubated overnight in a buffer containing 50 mM Tris-HCl, pH 7.6, 5 mM CaCl_2_, 1 μM ZnCl_2_, and 1% Triton X-100. Triton was used to remove SDS from the gel. Gels were then stained with Coomassie brilliant blue for 2–3 h and destained with 7% acetic acid or water. Gelatinolytic activity was detected as clear bands in the background of blue staining [ref.28].
