# Supplementary material for: Integrin αvβ3 and CD44 pathways in metastatic prostate cancer cells support osteoclastogenesis via a Runx2/Smad 5/receptor activator of NF-κB ligand signaling axis
Source: Mol Cancer. 2012 Sep 11;11:66. doi: 10.1186/1476-4598-11-66 (PMC3499378; doi:10.1186/1476-4598-11-66)
Supplement: Additional file 2 — Figure S2.Immunoblotting analysis for Smad 2, 3, 5 and 6 proteins in PC3 cells. About 50μg total cellular lysate protein was used for immunoblotting with antibodies to phospho-Smad (p-Smad) -2 (60kDa; lane 1), -3 (52 kDa; lane 2), -5 (60kDa; lane 3) and -6 (62kDa; lane 4). Blots were reprobed with an antibody to GAPDH after stripping. Phosphorylation of 2, 3, and 5 was observed in PC3 cells. However, Smad- 5 phosphorylation is significantly more than Smad-2 and 3 (lanes 1 and 2). Phosphorylation of Smad-6 is really negligible or not observed. [file 1476-4598-11-66-S2.doc]

Additional Figure 2


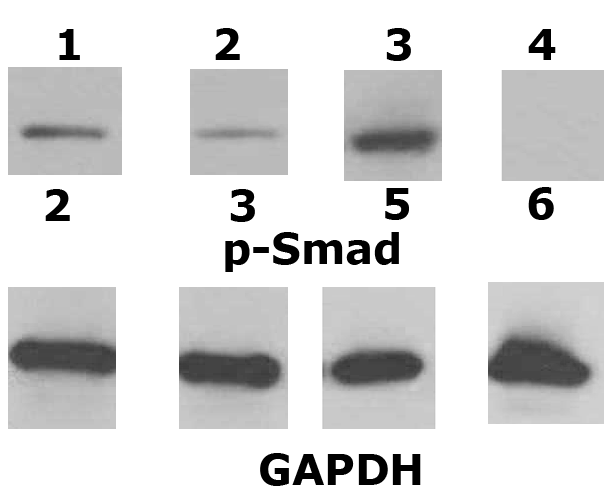


Additional figure 2

About 50µg total cellular lysate protein was used for immunoblotting with antibodies to phospho-Smad (p-Smad) -2 (60kDa; lane 1), -3 (52 kDa; lane 2), -5 (60kDa; lane 3) and -6 (62kDa; lane 4). Blots were reprobed with an antibody to GAPDH after stripping. Phosphorylation of 2, 3, and 5 was observed in PC3 cells. However, Smad- 5 phosphorylation is significantly more than Smad-2 and 3 (lanes 1 and 2). Phosphorylation of Smad-6 is really negligible or not observed.

**Method**

Gelatin zymography**:** Conditioned media collected from various PC3 cell lines were concentrated approximately 10-fold) with a centricon concentrator (Amicon, Beverly, MA). Ten micrograms of concentrated media protein in 10-20 μl were mixed with gel loading buffer with no reducing agent (βME or DTT) and incubated at RT for 10–15 min. SDS-PAGE containing 0.1% gelatin was used for electrophoresis. Samples were loaded without heating with sample buffer. After electrophoresis, gels were incubated overnight in a buffer containing 50 mM Tris-HCl, pH 7.6, 5 mM CaCl_2_, 1 μM ZnCl_2_, and 1% Triton X-100. Triton was used to remove SDS from the gel. Gels were then stained with Coomassie brilliant blue for 2–3 h and destained with 7% acetic acid or water. Gelatinolytic activity was detected as clear bands in the background of blue staining [ref.28].
